# Burnout Among Primary Care Practitioners and Staff in VA Clinics Using Virtual Contingency Staffing

**DOI:** 10.1001/jamanetworkopen.2025.18977

**Published:** 2025-07-03

**Authors:** Eric A. Apaydin, Caroline K. Yoo, Nicholas J. Jackson, Susan E. Stockdale, Danielle E. Rose

**Affiliations:** 1 Center for the Study of Healthcare Innovation, Implementation and Policy, VA Greater Los Angeles Healthcare System, Los Angeles, California; 2 Department of Medicine, David Geffen School of Medicine, University of California, Los Angeles; 3 RAND Corporation, Santa Monica, California; 4 Department of Psychiatry and Biobehavioral Sciences, David Geffen School of Medicine, University of California, Los Angeles

## Abstract

**Importance:**

Primary care practitioners (PCPs) and staff in Veterans Health Administration (VHA) clinics with staffing shortages have reported higher rates of burnout that may be associated with higher workloads. Introducing PCPs through the Clinical Research Hub (CRH) virtual contingency staffing program into these clinics may help reduce burnout but may also disrupt workflows and increase burnout.

**Objective:**

To understand how rates of burnout among VHA PCPs and staff vary by staffing level and CRH program use.

**Design, Setting, and Participants:**

This survey study used annual, repeated, cross-sectional VHA employee survey data from fiscal years 2018 to 2022 to examine associations between staffing and burnout before and after implementation of the CRH program.

**Exposure:**

Clinical Research Hub virtual contingency PCP visits.

**Main Outcome and Measures:**

The main outcome was burnout as measured using multilevel, mixed-effects logistic regression to estimate the association between health care system–level PCP staffing and individual-level PCP and staff burnout before and after implementation of the CRH program. An interaction term was used to test the association between program use and burnout in health care systems with full and less-than-full PCP staffing, controlling for PCP, staff, and health care system characteristics. Estimated marginal means of burnout were calculated from model results.

**Results:**

Survey responses from 134 640 PCPs and staff (53% younger than 49 years; 70% female) in 139 VHA health care systems were analyzed. From fiscal years 2018 to 2022, 38% of PCPs and staff experienced burnout, and CRH visits ranged from a median of 0 to 127.6 (IQR, 76.7-237.4) visits per 1000 patients at the health care system level. In estimations derived from the full model, the probability of burnout was higher in clinics without full PCP staffing before program implementation (34.3% [95% CI, 33.4%-35.2%] without full staffing vs 36.5% [95% CI, 35.3%-37.8%] with full staffing) and in the lowest tertile of CRH visits (37.4% [95% CI, 36.4%-38.4%] without full staffing vs 40.2% [95% CI, 38.3%-42.1%] with full staffing). However, burnout did not differ by staffing at higher levels of CRH visits.

**Conclusions and Relevance:**

In this survey study of VHA PCPs and staff, the association between low staffing and burnout was mitigated at higher levels of CRH program use, suggesting that contingency PCPs may alleviate high workload in short-staffed clinics.

## Key Points

**Question:**

Do rates of burnout vary among Veterans Health Administration (VHA) primary care practitioners (PCPs) and staff by staffing level and virtual contingency staffing program use?

**Findings:**

In this survey study of 134 640 PCPs and staff across 139 VHA clinics, working in clinics before the implementation of the virtual contingency staffing program or that were in the lowest tertile of program use was associated with a greater likelihood for burnout if the clinic did not have full PCP staffing. This association did not differ by PCP staffing level in the higher tertiles of program use.

**Meaning:**

These findings suggest that VHA virtual contingency staffing may alleviate high workload in short-staffed clinics.

## Teaser

This survey study examines rates of burnout among US Department of Veterans Affairs (VA) primary care practitioners and staff by staffing level and virtual contingency staffing program use.

## Introduction

By 2037, it is estimated that the US will be short nearly 90 000 primary care physicians, or just more than 25% of the primary care physician workforce.^1^ Increases in the number of nurse practitioners and physician assistants may not be enough to fulfill the demand for primary care in many parts of the country.^2,3^ Like private health care systems, the Veterans Health Administration (VHA) also faces shortages of primary care practitioners (PCPs) (physicians, nurse practitioners, and physician assistants) and staff (registered nurses, nursing assistants, clerks, and other professionals). Virtual care, which expanded considerably during the COVID-19 pandemic,^4,5^ may help relieve some of these shortages^6^ by allowing patients and PCPs to meet by phone or videoconferencing from any location.^7^ At the same time, burnout among PCPs^8,9,10^ and staff^11,12^ has grown since the pandemic^11,13,14^ and is now recognized as a national crisis.^15,16^ Virtual care may be able to alleviate both shortages and burnout by virtually redistributing workload across VHA clinics.

In 2019, the VHA launched the national Clinical Resource Hub (CRH) virtual contingency staffing program in primary care, which allowed patients who use clinics with fewer PCPs to use virtual care to see a PCP at a more well-staffed hospital or clinic.^17^ The program consists of hubs located in each VHA region, which provide up to 2 years of staffing support for primary care outpatient services at understaffed clinic spoke sites within the same region.^18^ This staffing support consists mainly of virtual care from hub sites to patients at understaffed spoke sites. The CRH program was intended to improve patient access to care in sites with primary care staffing vacancies. There is evidence that the program increased primary care virtual visits and reduced emergency department visits and hospital stays among patients who received care in sites using CRHs vs patients receiving care at comparison sites.^19^

The CRH program may reduce PCP shortages by connecting patients and PCPs from disparate locations, remedying imbalances in supply and demand. The program may also reduce PCP and staff burnout by decreasing the demand of local workload and increasing the resource of virtual PCPs. However, the program could also increase burnout by increasing local demands due to the complicated nature of the program or its disruptions of local workflow. Several theoretical models of burnout have informed our hypotheses about the association between CRHs and burnout. The Areas of Worklife Survey (AWS) model theorizes that high workload directly contributes to increased burnout.^20^ On the other hand, the Job Demands-Resources (JD-R) model theorizes that job demands (eg, workload) and resources (eg, staffing levels) compete to result in higher or lower burnout.

The associations among the CRH program, workload, and burnout in VHA PCPs and staff have not yet been studied. We hypothesized that the CRH program reduced burnout among PCPs and staff who worked in the health care systems where it was implemented but only if their systems experienced PCP shortages. To analyze the associations among CRH program use, PCP shortages, and PCP and staff burnout, we examined VHA survey and administrative data from fiscal years 2018 to 2022.

## Methods

### Design

This survey study analyzed individual- and health care system–level data drawn from repeated survey cross sections matched with administrative records. Health care systems were operationalized as hospitals and their associated outpatient clinics. Individual-level survey data (eg, burnout, demographics) were matched with health care system–level administrative data (eg, CRH use, staffing levels). This work was designated nonresearch and not subject to review by an institutional review board since it was performed as a quality improvement evaluation under the terms of a signed attestation of nonresearch from the VHA Office of Primary Care. This documentation ensures that work not performed under a human participants protocol is part of institutionally sanctioned quality improvement activities. Informed consent was not applicable to this analysis as it was a secondary analysis of survey data and designated as nonresearch. Participant disclosures complied with the American Association of Public Opinion Research (AAPOR) Transparency Initiative.^21^

### Data Sources and Sample

We used the VHA All Employee Survey (AES),^22^ an anonymous survey of employee experience^23^ conducted by email every year in June. In 2020, the survey was administered in September due to pandemic-related delays. The AES is conducted by the VHA National Center for Organization Development and is administered to all full- and part-time VHA employees. Supervisors email workgroup codes to their employees in each particular job or functional workgroup, and employees use those codes to log into the survey. The data are always unweighted, but they are checked against human resources administrative data and for data entry irregularities. Data failing multiple quality checks (eg, very long time to complete, more responses in a workgroup than employees, very few questions answered) are removed. The codebook for the most recent iteration of the AES is available online.^24^

Data on COVID-19 burden was acquired from the COVID Shared Data Resource,^25^ a VHA-wide data source on COVID-19 testing, diagnosis, resource use, and mortality. All other data were derived from the VHA Corporate Data Warehouse,^26^ a national repository of clinical, enrollment, benefit, administrative, and financial data.

For this study, we analyzed responses from primary care personnel across VHA health care systems from fiscal years 2018 to 2022, including PCPs, registered nurses, clinical associates (ie, licensed vocational nurses, licensed practical nurses), administrative associates (ie, administrative staff, clerks), other professional staff members (eg, pharmacists, social workers), and responses without specified team roles. These professionals (PCPs and other staff) comprise the VHA Patient Aligned Care Team (PACT) teamlet that works together daily. The PACT is the VHA implementation of the Patient-Centered Medical Home.

### Variables

At the individual level, we include data from the AES on burnout, demographics, and perceived workload. Burnout is the subject of our study, and demographics and workload have been associated with burnout in previous work on VHA primary care.^27^ Data on race and ethnicity were self-reported (Hispanic, non-Hispanic Asian, non-Hispanic Black, non-Hispanic Pacific Islander, non-Hispanic White, and non-Hispanic other [ie, all respondents who reported other race and non-Hispanic ethnicity) and included because race and ethnicity were associated with burnout in previous studies of VHA primary care.^27,28,29^

At the health care system level, we include variables for CRH use, PCP and teamlet staffing, health care system complexity, COVID-19 testing burden, rurality, and geographic region. Again, CRH is the subject of our study, and the other health care system–level variables were associated with burnout or used as controls in previous work on VHA primary care.^27,28,30,31^ The sole exception is the study by O’Shea et al,^32^ which was a novel measure of PCP staffing that we used here instead of the more conventional measure of panel overcapacity. Additional details on all variables, including item and response descriptions, coding, and sources, are provided in the eMethods in Supplement 1.

### Statistical Analysis

Multilevel mixed-effects logistic regression models were used to analyze the associations between individual-level burnout, health care system–level CRH use, and health care system–level CRH and primary care staffing interactions, controlling for individual-level demographics and perceptions of workload, and health care system–level fixed effects of PCP and teamlet staffing, complexity, patient rurality, census region, and COVID-19 burden. Random intercepts were included by health care system to account for clustering in an intercept-only model (intraclass correlation coefficient, 0.41; 95% CI, 0.28-0.59). Interactions were used to evaluate the combined associations of CRH use and staffing with burnout. In our first supplementary analysis, interaction terms were removed and only main effects presented. We also present supplementary subgroup analyses by profession.

For all models, we used Stata, version 18.0 (StataCorp LLC). Significance was set at a 2-tailed α of .05. Stata commands melogit and margins were used for mixed-effects logistics regression models and estimate margins, respectively.

In compliance with Executive Order 14168, “Defending Women From Gender Ideology Extremism and Restoring Biological Truth to the Federal Government,” we only report male and female responses for sex from the AES and have marked other categories as missing. These responses of nonmale and nonfemale identity were only collected for the AES during fiscal year 2022. We also included missing responses for all other AES variables in our descriptive statistics and analytic models. Odds ratios (ORs) for these categories are not reported for our models, and respondents with missing burnout data were not included in our study.

## Results

Among 134 640 PCPs and staff (including 22 362 PCPs, 32 287 registered nurses, 28 020 clinical associates, 12 510 administrative associates, 41 553 other professional staff members, and 365 staff members without specified team roles) across 139 health care systems from fiscal years 2018 to 2022, the rate of burnout was 38% (Table 1). Most respondents were younger than 49 years (53% vs 45% aged ≥50 years [4% missing data]), female (70% vs 25% male [5% missing data]), non-Hispanic White (52% compared with 9% Hispanic, 9% non-Hispanic Asian, 20% non-Hispanic Black, 1% non-Hispanic Pacific Islander, and 4% non-Hispanic other [5% missing data]), and had 10 years or less of VHA tenure (68% vs 30% with ≥20 years [2% missing data]). No profession made up a majority of the sample, and most respondents reported that their workload was reasonable (74%).

**Table 1.** Characteristics of VHA PCPs and Staff, Fiscal Years 2018-2022 (N = 134 640)

Characteristic	PCPs and staff, No. (%)
Age, y	
<30	6365 (5)
30-39	28 495 (21)
40-49	36 372 (27)
50-59	40 460 (30)
≥60	20 595 (15)
Missing	4810 (4)
Sex	
Male	33 908 (25)
Female	96 550 (70)
Missing	6639 (5)
Race and ethnicity	
Hispanic	12 993 (9)
Non-Hispanic Asian	11 744 (9)
Non-Hispanic Black	27 595 (20)
Non-Hispanic Pacific Islander	1227 (1)
Non-Hispanic White	70 858 (52)
Non-Hispanic other^a^	4985 (4)
Missing	7695 (6)
VHA tenure, y	
<1	17 295 (13)
1-5	45 784 (33)
6-10	30 684 (22)
11-20	29 789 (22)
>20	10 375 (8)
Missing	3170 (2)
PACT team role	
PCP	22 362 (16)
Registered nurse	32 287 (24)
Clinical associate	28 020 (20)
Administrative associate	12 510 (9)
Other PACT professional^b^	41 553 (30)
Missing	365 (0)
Reasonable workload	
Neutral or agree	100 994 (74)
Disagree	34 776 (25)
Missing	1367 (1)
Burnout	
No	83 383 (62)
Yes	51 773 (38)

Abbreviations: PACT, Patient Aligned Care Team; PCP, primary care practitioner; VHA, Veterans Health Administration.

^a^ The Non-Hispanic other group included all respondents who self-reported other race and non-Hispanic ethnicity.

^b^ Including, eg, clinical pharmacists or social workers.

Clinical research hub visits started in fiscal year 2020 and ranged from a median (IQR) of 4.1 (0.2-26.1) per 1000 primary care visits for the middle tertile of health care systems in that year to a median (IQR) of 127.6 (76.7-237.4) per 1000 primary care visits for the highest tertile (Table 2). In 2021, CRH visits ranged from a median (IQR) of 14.6 (8.1-26.8) to 104.4 (74.2-182.8) per 1000 primary care visits between the highest 2 tertiles, and in 2022, they ranged from 6.1 (0.6-19.5) to 112.3 (74.9-212.3) per 1000 primary care visits between these 2 tertiles. The lowest tertile had no CRH visits in all 4 years.

**Table 2.** Characteristics of Health Care–System Level Variables, Fiscal Years 2018-2022

Characteristic	Fiscal year				
	2018 (n = 138)	2019 (n = 139)	2020 (n = 139)	2021 (n = 139)	2022 (n = 139)
Ranked tertile of CRH visits per 1000 primary care visits, median (IQR)					
Before implementation	0	0	0	0	0
First (lowest)	0	0	0	0	0
Second (middle)	0	0	4.1 (0.2-26.1)	14.6 (8.1-26.8)	6.1 (0.6-19.5)
Third (highest)	0	0	127.6 (76.7-237.4)	104.4 (74.2-182.8)	112.3 (74.9-212.3)
Health care system–level physician staffing, No. (%)					
<1.2 (Not fully staffed)	35 (25)	36 (26)	23 (17)	20 (14)	20 (14)
≥1.2 (Fully staffed)	103 (75)	103 (74)	116 (83)	119 (86)	119 (86)
Health care system–level percentage of core teamlets with full staffing, No. (%)^a^					
<50	70 (51)	65 (47)	73 (53)	76 (55)	75 (54)
≥50	68 (49)	74 (53)	66 (47)	63 (46)	64 (46)
Facility complexity, No. (%)					
High	39 (28)	39 (28)	39 (28)	39 (28)	39 (28)
Medium	52 (38)	53 (38)	53 (38)	44 (32)	44 (32)
Low	47 (34)	47 (34)	47 (34)	55 (40)	55 (40)
Missing	0	0	0	1 (1)	1 (1)
Patients residing in rural area, median (IQR), %	39.1 (20.5-53.3)	39.2 (20.5-53.1)	39.4 (20.6-53.4)	39.4 (20.8-53.1)	39.0 (20.6-52.5)
Census region, No. (%)					
Northeast	30 (22)	30 (22)	30 (22)	30 (22)	30 (22)
Midwest	41 (30)	41 (29)	41 (29)	41 (29)	41 (29)
South	36 (26)	36 (26)	36 (26)	36 (26)	36 (26)
West	31 (22)	32 (23)	32 (23)	32 (22)	32 (23)
COVID-19 tests per 1000 patients, median (IQR), No.	0	0	48.3 (39.8-63.1)	152.7 (123.4-183.5)	116.6 (97.8-139.8)

Abbreviation: CRH, Clinical Resources Hub.

^a^ Full core teamlet staffing is at least 3 staff members per primary care practitioner.

Across all 4 years, 74% to 86% of health care systems had full PCP staffing, and 46% to 53% had full teamlet staffing (≥3 team members per PCP) (Table 1). Of health care systems, 28% had high facility complexity across all 4 years, and patient rurality ranged from a median (IQR) of 39.0% (20.6%-52.5%) to 39.4% (20.6%-53.4%). Most health care systems were located in the Midwest (range, 29%-30%), and COVID-19 tests ranged from a median (IQR) of 48.3 (39.8-63.1) to 152.7 (123.4-183.5) per 1000 patients during the pandemic (2020-2022).

In fully adjusted models with interaction terms, burnout was higher in health care systems without full staffing before CRH implementation and among systems that did not implement CRH (Figure). The estimated probability of burnout was higher (36.5%; 95% CI, 35.3%-37.8%) for health care workers in systems that were not fully staffed before CRH implementation compared with those in fully staffed health care systems during that same period (34.3%; 95% CI, 33.4%-35.2%) (Table 3). Burnout probability was also higher in health care systems without full staffing that did not implement CRH (first [lowest] tertile) (40.2%; 95% CI, 38.3%-42.1%) compared with those with full staffing (37.4%; 95% CI, 36.4%-38.4%). Burnout did not vary by PCP staffing in health care systems that used the CRH program.

**Figure.**
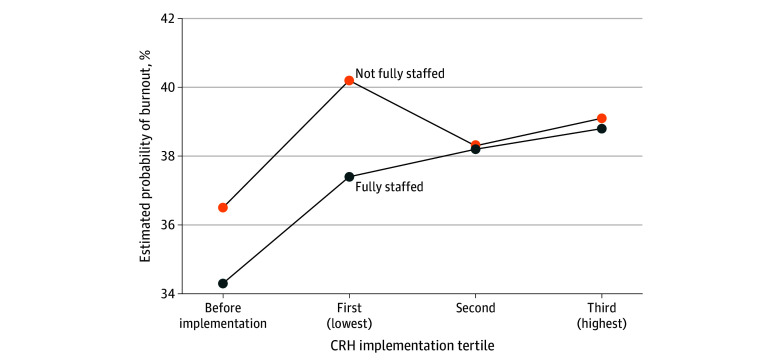
Estimated Probability of Burnout by Clinical Resource Hub (CRH) Implementation Percentile and Staffing Level, Fiscal Years 2018-2022

**Table 3.** Estimated Probabilities of Burnout Among VHA PCPs and Staff Using Multilevel Mixed-Effects Logistic Regression, Fiscal Years 2018-2022

Ranked tertile of CRH visits per 1000 primary care visits	% (95% CI)	
	Less than full PCP staffing	Full PCP staffing
Before implementation	36.5 (35.3-37.8)	34.3 (33.4-35.2)
First (lowest)	40.2 (38.3-42.1)	37.4 (36.4-38.4)
Second (middle)	38.3 (36.7-40.0)	38.2 (37.2-39.2)
Third (highest)	39.1 (37.3-40.9)	38.8 (37.8-39.9)

Abbreviations: CRH, Clinical Resources Hub; PCP, primary care practitioner; VHA, Veterans Health Administration.

The probability of burnout was slightly lower among health care workers in systems with at least 50% of teamlets with full staffing (OR, 0.96; 95% CI, 0.93-0.99) compared with workers in systems with less than 50% of fully staffed teams (Table 4). Registered nurses (OR, 0.67; 95% CI, 0.65-0.71), clinical associates (OR, 0.63; 95% CI, 0.60-0.65), administrative associates (OR, 0.77; 95% CI, 0.73-0.82), and other PACT professionals (OR, 0.66; 95% CI, 0.64-0.69) all were less likely to experience burnout than PCPs. The probability of burnout was also lower for older respondents (aged ≥60 years: OR, 0.42; 95% CI, 0.39-0.45). Female respondents were slightly less likely to experience burnout than male respondents (OR, 0.95; 95% CI, 0.92-0.98). Non-Hispanic Asian (OR, 0.89; 95% CI, 0.85-0.94) and non-Hispanic Black (OR, 0.90; 95% CI, 0.87-0.93) PCPs and staff were less likely to experience burnout than non-Hispanic White PCPs and staff, but non-Hispanic respondents identifying their race and ethnicity as other were more likely to experience burnout (OR, 1.14; 95% CI, 1.07-1.22). Burnout was highest among respondents with 12 to 20 years of VHA tenure (OR, 2.32; 95% CI, 2.21-2.44), and respondents in the Northeast (OR, 0.87; 95% CI, 0.81-0.94) and South (OR, 0.92; 95% CI, 0.86-0.99) were less likely to experience burnout than those in the West. The PCPs and staff who disagreed that their workload was reasonable (OR, 7.69; 95% CI, 7.47-7.91) were much more likely to experience burnout than those who agreed or were neutral. Each additional COVID-19 test per 1000 patients only slightly increased the odds of burnout (OR, 1.001; 95% CI, 1.001-1.002), which implies a meaningful increase in odds of burnout at the health care systems with the highest number of COVID-19 tests. Health care system complexity had no association with burnout.

**Table 4.** Odds of Burnout Among VHA PCPs and Staff Using Multilevel Mixed-Effects Logistic Regression, Fiscal Years 2018-2022

Characteristic	OR (95% CI)
Ranked tertile of CRH visits per 1000 primary care visits	
Before implementation	1 [Reference]
First (lowest)	1.14 (1.08-1.21)^a^
Second (middle)	1.18 (1.12-1.25)^a^
Third (highest)	1.22 (1.15-1.29)^a^
Health care system–level PCP staffing	
<1.2 (Not fully staffed)	1.10 (1.04-1.17)^a^
≥1.2 (Fully staffed)	1 [Reference]
CRH program use by health care system–level PCP staffing	
Before implementation and not fully staffed	1 [Reference]
First tertile and not fully staffed	1.03 (0.93-1.12)
Second tertile and not fully staffed	0.91 (0.84-0.99)^b^
Third tertile and not fully staffed	0.92 (0.84-1.00)
Health care system–level percentage of core teamlets with full staffing^c^	
<50	1 [Reference]
≥50	0.96 (0.93-0.99)^b^
PACT team role	
PCP	1 [Reference]
Registered nurse	0.67 (0.65-0.71)^a^
Clinical associate	0.63 (0.60-0.65)^a^
Administrative associate	0.77 (0.73-0.82)^a^
Other PACT professional^d^	0.66 (0.64-0.69)^a^
Age, y	
<30	1 [Reference]
30-39	0.79 (0.74-0.84)^a^
40-49	0.63 (0.60-0.67)^a^
50-59	0.53 (0.49-0.56)^a^
≥60	0.42 (0.39-0.45)^a^
Sex	
Female	0.95 (0.92-0.98)^a^
Male	1 [Reference]
Race and ethnicity	
Hispanic	0.99 (0.95-1.04)
Non-Hispanic Asian	0.89 (0.85-0.94)^a^
Non-Hispanic Black	0.90 (0.87-0.93)^a^
Non-Hispanic Pacific Islander	1.03 (0.90-1.18)
Non-Hispanic White	1 [Reference]
Non-Hispanic other^e^	1.14 (1.07-1.22)^a^
VHA tenure, y	
<1	1 [Reference]
1-5	1.80 (1.72-1.88)^a^
6-10	2.21 (2.10-2.31)^a^
12-20	2.32 (2.21-2.44)^a^
>20	2.21 (2.08-2.36)^a^
Reasonable workload	
Neutral or agree	1 [Reference]
Disagree	7.69 (7.47-7.91)^a^
Facility complexity	
High	1 [Reference]
Medium	0.95 (0.90-0.99)^b^
Low	1.05 (0.98-1.13)
Percentage of patients residing in rural area	1.00 (1.00-1.00)
Census region	
Northeast	0.87 (0.81-0.94)^a^
Midwest	0.94 (0.88-1.02)
South	0.92 (0.86-0.99)^b^
West	1 [Reference]
No. of COVID-19 tests per 1000 patients	1.001 (1.001-1.002)^a^

Abbreviations: CRH, Clinical Resources Hub; OR, odds ratio; PACT, Patient Aligned Care Team; PCP, primary care practitioner; VHA, Veterans Health Administration.

^a^ *P* < .001.

^b^ *P* < .05.

^c^ Full core teamlet staffing is at least 3 staff per PCP.

^d^ Including, eg, clinical pharmacists or social workers.

^e^ The Non-Hispanic other group included all respondents who self-reported other race and non-Hispanic ethnicity.

In supplementary analyses without interaction terms, burnout was higher among all health care systems without full PCP staffing (OR, 1.07; 95% CI, 1.02-1.12) compared with those with full staffing (eTable 1 in Supplement 1). Burnout also rose after CRH implementation during the pandemic. Burnout was higher among health care systems with low (OR, 1.14; 95% CI, 1.08-1.20), middle (OR, 1.15; 95% CI, 1.10-1.21), and high (OR, 1.19; 95% CI, 1.13-1.25) tertiles of CRH use compared with before implementation. In supplementary subgroup analyses by profession (eTables 2-17 and eFigures 1-8 in Supplement 1), differences in burnout by PCP staffing level only appeared for all PCPs and physicians (lowest tertile of CRH use), clinical and administrative associates (before implementation of CRH), and other PACT professionals (both before implementation and lowest tertile of CRH use).

## Discussion

This survey study shows high rates of burnout among PCPs and staff during fiscal years 2018 to 2022, but these rates varied by health care system CRH use, PCP and PACT team member staffing levels, and perceived workload. Health care workers in systems without full PCP staffing had a 7% higher risk of burnout than those with full staffing. Conversely, the use of the CRH program in a health care system was associated with a 13% to 19% higher likelihood of burnout. However, when we tested for potential interactions, the use of CRHs interacted with staffing levels to reduce burnout. Before CRH implementation, the estimated probability of burnout for individual PCPs and staff was 2.2% higher in health care systems without full PCP staffing. After program implementation, among systems that did not use CRH, this difference rose to 3.1%. There was no difference in burnout between health care systems that used the CRH program regardless of their PCP staffing levels. Burnout rose after CRH implementation in all health care systems, but this period was concurrent with the pandemic. In addition, health care workers with a workload perceived to be unreasonable had more than 7 times the rates of burnout as those who perceived their workloads as reasonable. As differences in burnout by PCP staffing level were eliminated after the introduction of CRH, we believe that the program may have filled in the staffing gaps present in health care systems with fewer PCPs, thereby reducing PCP and staff workload and their rates of burnout. The program was observed to have no association with burnout if enough PCPs were already available to handle the system’s workload.

Workload is a core feature of 2 major models of burnout, including the AWS^20^ and JD-R^33,34^ models. The AWS model explicitly links reasonable workload to lower burnout, while the JD-R model characterizes workload more generally as a job demand, which is a category that also includes other job stressors such as interpersonal conflict and the cognitive complexity of one’s work. Staffing is not explicitly defined in the AWS model, but qualitative work by Leiter and Maslach^20^ showed that complaints about poor staffing were negatively correlated with the model’s reasonable workload score. On the other hand, the JD-R model categorizes staffing as a job resource,^34^ which competes with job demands to raise or lower worker burnout.

There is some evidence that inadequate staffing^30,31,35^ and high workload^27,31^ are associated with burnout in VHA primary care. There are also reviews that have shown a possible connection among burnout, staffing, and workload in health care systems outside the VHA.^15,36^ The CRH program was designed to use hub sites within a VHA region to accommodate unexpected, short-term changes in staffing at spoke sites in the same region.^18^ However, many VHA health care system leaders are skeptical of relying on the program,^37^ so longer-term solutions may be needed in systems with persistent shortages. It is possible that the CRH virtual contingency staffing program may improve staffing at spoke health care systems that are understaffed, thereby reducing the workload of the other onsite PCPs and staff. The program is not associated with burnout in fully staffed systems possibly because non-CRH patients in these systems are able to see their PCPs without the need for outside virtual care. The CRH program may simply draw in more patients for care in these systems.

Use of CRHs also was observed to alleviate burnout only among physicians, clinical associates, administrative associates, and other PACT professionals. Notably, this relief excluded nurses and nurse practitioners, but it is unclear why. The VHA generally employs more clinical than administrative staff across professions compared with the private sector.^38^ However, VHA primary care nursing may be particularly well staffed and resistant to burnout.

Our results validate both the AWS and JD-R models of burnout. Higher workload or increased job demands, as operationalized by increased perceptions of an unreasonable workload, was associated with a higher probability of burnout. However, increased job resources, as operationalized by increased CRH use when PCP staffing was inadequate, offset those job demands or reduced workload and stopped the increase in burnout.

The VHA primary care panel sizes are largely fixed by PCP type, staffing, resources, and patient complexity,^39^ so health care systems with adequate staffing should be able to meet patient demand for appointments and manage their workload. Appointments in VHA primary care may be resource constrained rather than demand constrained,^40^ meaning that patients within a panel will always take up all of the available appointment times^41^ and that fully staffed teams should be able to accommodate that workload without an increase in burnout.^31^ The CRH virtual contingency staffing program may act as a temporary resource expansion for short-term staffing needs, reducing the workload and burnout of PCPs and staff in understaffed health care systems but having little influence on burnout in systems with full staffing.

Our results suggest that virtual care contingency staffing programs such as CRHs could be rolled out in non-VHA primary care settings to not only benefit patients and reduce costs but also reduce PCP and staff burnout. The CRH program increased the use of virtual primary care since its inception,^42^ and there is some evidence that the program has maintained access to care^17,43^ and reduced emergency department visits and hospitalizations.^19^ There is also some evidence that virtual primary care outside the VHA has similar quality, cost, and access benefits according to a cross-country survey of PCPs^44^ and a recent systematic review.^45^ The CRH program was not associated with increased burnout despite the potential for disrupting local workflows (eg, local nurses or medical assistants may not be able to reschedule or move CRH appointments) by adding hub telehealth PCPs to spoke sites. It is possible that the program was self-contained enough to not affect non-CRH appointments at a local site but any disarray related to the program may have been offset by the reduction in burnout associated with reduced workload demands. The CRH program could be adapted for use in other large, multiclinic, private sector health care systems that have a geographic imbalance of patients and PCPs. These systems should weigh the costs of the technological infrastructure needed for virtual staffing against the costs of higher salaries to entice PCPs to physically relocate when considering the implementation of a virtual contingency staffing program.

### Strengths and Limitations

This study had many strengths, including the use of several years of survey and administrative data from a national health care system and a repeated cross-sectional, before-and-after design. However, this study also had several limitations. First, the outcome, main variable, and covariates were measured at mixed levels, including individual-level burnout and health care system–level CRH use. However, there may still be meaningful connections among these mixed-level variables, as system-level CRH use may influence the demand for care and, thus, the burnout of local health care workers who do not use CRHs. Second, most PCPs and staff reported a reasonable workload, so our results may not be generalizable to other health care systems or clinics in which the workload is perceived as unreasonable by most. Third, we did not use a direct measure of workload, only a survey question about the reasonableness of respondents’ workloads. However, workload reasonableness may not be completely objective as different PCPs and staff can accommodate different levels of work, so self-perceived workload may reflect more how one keeps up with a discrete workload. Fourth, CRH implementation was concurrent with the onset of the COVID-19 pandemic, so outcomes associated with the program may have been obscured by the unusually high burnout during this period. More recent CRH data will be needed to extend our analyses to the postpandemic period. Fifth, our analysis used a repeated cross-sectional design, which cannot be used to infer causal direction, so it is theoretically possible that poor PCP staffing and high health care worker burnout induced more CRH use rather than a CRH alleviating each. Such a causal direction is unlikely as there seems to be a temporal and dose-response relationship between CRH use and lower burnout contingent on poor staffing.

## Conclusions

This survey study shows that the CRH virtual contingency staffing program was associated with lower burnout among PCPs and health care workers in health care systems with poor PCP staffing but not in health care systems with full staffing. Virtual contingency staffing programs should be explored further, as they may help alleviate temporary PCP staffing deficiencies, thereby reducing the workload and burnout of overworked PCPs and staff. Burnout and staffing shortages are multifaceted and challenging problems for the delivery of primary care, but implementing virtual contingency staffing may offer short-term relief for both.

## Notes

Supplement 1.
eMethods.

eReferences.
**eTable 1.** Odds Ratios and 95% CIs of Burnout Among VHA Primary Care Practitioners and Staff Using Multilevel Mixed-Effects Logistic Regression (Main Effects Only), Fiscal Years 2018-2022 **eTable 2.** Odds Ratios and 95% CIs of Burnout Among VHA Primary Care Practitioners and Staff Using Multilevel Mixed-Effects Logistic Regression (Practitioner [Physicians, Nurse Practitioners, and Physician Assistants] Only), 2018-2022 **eTable 3.** Estimated Probabilities of Burnout Among VHA Primary Care Practitioners and Staff Using Multilevel Mixed-Effects Logistic Regression (Practitioners Only), Fiscal Years 2018-2022 **eFigure 1.** Estimated Probability of Burnout by Clinical Resource Hub (CRH) Implementation Percentile and Staffing Level (Practitioners Only), Fiscal Years 2018-2022 **eTable 4.** Odds Ratios and 95% CIs of Burnout Among VHA Primary Care Practitioners and Staff Using Multilevel Mixed-Effects Logistic Regression (Physicians Only), 2018-2022 **eTable 5.** Estimated Probabilities of Burnout Among VHA Primary Care Practitioners and Staff Using Multilevel Mixed-Effects Logistic Regression (Physicians Only), Fiscal Years 2018-2022 **eFigure 2.** Estimated Probability of Burnout by Clinical Resource Hub (CRH) Implementation Percentile and Staffing Level (Physicians Only), Fiscal Years 2018-2022 **eTable 6.** Odds Ratios and 95% CIs of Burnout Among VHA Primary Care Practitioners and Staff Using Multilevel Mixed-Effects Logistic Regression (Nurse Practitioners Only), 2018-2022 **eTable 7.** Estimated Probabilities of Burnout Among VHA Primary Care Practitioners and Staff Using Multilevel Mixed-Effects Logistic Regression (Nurse Practitioners Only), Fiscal Years 2018-2022 **eFigure 3.** Estimated Probability of Burnout by Clinical Resource Hub (CRH) Implementation Percentile and Staffing Level (Nurse Practitioners Only), Fiscal Years 2018-2022 **eTable 8.** Odds Ratios and 95% CIs of Burnout Among VHA Primary Care Practitioners and Staff Using Multilevel Mixed-Effects Logistic Regression (Physician Assistants Only), 2018-2022 **eTable 9.** Estimated Probabilities of Burnout Among VHA Primary Care Practitioners and Staff Using Multilevel Mixed-Effects Logistic Regression (Physician Assistants Only), Fiscal Years 2018-2022 **eFigure 4.** Estimated Probability of Burnout by Clinical Resource Hub (CRH) Implementation Percentile and Staffing Level (Physician Assistants Only), Fiscal Years 2018-2022 **eTable 10.** Odds Ratios and 95% CIs of Burnout Among VHA Primary Care Practitioners and Staff Using Multilevel Mixed-Effects Logistic Regression (Registered Nurses Only), 2018-2022 **eTable 11.** Estimated Probabilities of Burnout Among VHA Primary Care Practitioners and Staff Using Multilevel Mixed-Effects Logistic Regression (Registered Nurses Only), Fiscal Years 2018-2022 **eFigure 5.** Estimated Probability of Burnout by Clinical Resource Hub (CRH) Implementation Percentile and Staffing Level (Registered Nurses Only), Fiscal Years 2018-2022 **eTable 12.** Odds Ratios and 95% CIs of Burnout Among VHA Primary Care Practitioners and Staff Using Multilevel Mixed-Effects Logistic Regression (Clinical Associates Only), 2018-2022 **eTable 13.** Estimated Probabilities of Burnout Among VHA Primary Care Practitioners and Staff Using Multilevel Mixed-Effects Logistic Regression (Clinical Associates Only), Fiscal Years 2018-2022 **eFigure 6.** Estimated Probability of Burnout by Clinical Resource Hub (CRH) Implementation Percentile and Staffing Level (Clinical Associates Only), Fiscal Years 2018-2022 **eTable 14.** Odds Ratios and 95% CIs of Burnout Among VHA Primary Care Practitioners and Staff Using Multilevel Mixed-Effects Logistic Regression (Administrative Associates Only), 2018-2022 **eTable 15.** Estimated Probabilities of Burnout Among VHA Primary Care Practitioners and Staff Using Multilevel Mixed-Effects Logistic Regression (Administrative Associates Only), Fiscal Years 2018-2022 **eFigure 7.** Estimated Probability of Burnout by Clinical Resource Hub (CRH) Implementation Percentile and Staffing Level (Administrative Associates Only), Fiscal Years 2018-2022 **eTable 16.** Odds Ratios and 95% CIs of Burnout Among VHA Primary Care Practitioners and Staff Using Multilevel Mixed-Effects Logistic Regression (Other PACT Professionals Only), 2018-2022 **eTable 17.** Estimated Probabilities of Burnout Among VHA Primary Care Practitioners and Staff Using Multilevel Mixed-Effects Logistic Regression (Other PACT Professionals Only), Fiscal Years 2018-2022 **eFigure 8.** Estimated Probability of Burnout by Clinical Resource Hub (CRH) Implementation Percentile and Staffing Level (Other PACT Professionals Only), Fiscal Years 2018-2022

Supplement 2.
Data Sharing Statement
