# Supplementary material for: Burnout Among Primary Care Practitioners and Staff in VA Clinics Using Virtual Contingency Staffing
Source: JAMA Netw Open. 2025 Jul 3;8(7):e2518977. doi: 10.1001/jamanetworkopen.2025.18977 (PMC12232184; doi:10.1001/jamanetworkopen.2025.18977)
Supplement: Supplement 1. — eMethods. eReferences. eTable 1. Odds Ratios and 95% CIs of Burnout Among VHA Primary Care Practitioners and Staff Using Multilevel Mixed-Effects Logistic Regression (Main Effects Only), Fiscal Years 2018-2022 eTable 2. Odds Ratios and 95% CIs of Burnout Among VHA Primary Care Practitioners and Staff Using Multilevel Mixed-Effects Logistic Regression (Practitioner [Physicians, Nurse Practitioners, and Physician Assistants] Only), 2018-2022 eTable 3. Estimated Probabilities of Burnout Among VHA Primary Care Practitioners and Staff Using Multilevel Mixed-Effects Logistic Regression (Practitioners Only), Fiscal Years 2018-2022 eFigure 1. Estimated Probability of Burnout by Clinical Resource Hub (CRH) Implementation Percentile and Staffing Level (Practitioners Only), Fiscal Years 2018-2022 eTable 4. Odds Ratios and 95% CIs of Burnout Among VHA Primary Care Practitioners and Staff Using Multilevel Mixed-Effects Logistic Regression (Physicians Only), 2018-2022 eTable 5. Estimated Probabilities of Burnout Among VHA Primary Care Practitioners and Staff Using Multilevel Mixed-Effects Logistic Regression (Physicians Only), Fiscal Years 2018-2022 eFigure 2. Estimated Probability of Burnout by Clinical Resource Hub (CRH) Implementation Percentile and Staffing Level (Physicians Only), Fiscal Years 2018-2022 eTable 6. Odds Ratios and 95% CIs of Burnout Among VHA Primary Care Practitioners and Staff Using Multilevel Mixed-Effects Logistic Regression (Nurse Practitioners Only), 2018-2022 eTable 7. Estimated Probabilities of Burnout Among VHA Primary Care Practitioners and Staff Using Multilevel Mixed-Effects Logistic Regression (Nurse Practitioners Only), Fiscal Years 2018-2022 eFigure 3. Estimated Probability of Burnout by Clinical Resource Hub (CRH) Implementation Percentile and Staffing Level (Nurse Practitioners Only), Fiscal Years 2018-2022 eTable 8. Odds Ratios and 95% CIs of Burnout Among VHA Primary Care Practitioners and Staff Using Multilevel Mixed-Effects Logistic Regres [file jamanetwopen-e2518977-s001.pdf]

Supplemental Online Content

Apaydin EA, Yoo CK, Jackson NJ, Stockdale SE, Rose DE. Burnout among primary care practitioners and staff in VA clinics using virtual contingency staffing. *JAMA Netw Open*. 2025;8(7):e2518977. doi:10.1001/jamanetworkopen.2025.18977

eMethods

eReferences

eTable 1. Odds Ratios and 95% CIs of Burnout Among VHA Primary Care Practitioners and Staff Using Multilevel Mixed-Effects Logistic Regression (Main Effects Only), Fiscal Years 2018-2022

eTable 2. Odds Ratios and 95% CIs of Burnout Among VHA Primary Care Practitioners and Staff Using Multilevel Mixed-Effects Logistic Regression (Practitioner [Physicians, Nurse Practitioners, and Physician Assistants] Only), 2018-2022

eTable 3. Estimated Probabilities of Burnout Among VHA Primary Care Practitioners and Staff Using Multilevel Mixed-Effects Logistic Regression (Practitioners Only), Fiscal Years 2018-2022

eFigure 1. Estimated Probability of Burnout by Clinical Resource Hub (CRH) Implementation Percentile and Staffing Level (Practitioners Only), Fiscal Years 2018-2022

eTable 4. Odds Ratios and 95% CIs of Burnout Among VHA Primary Care Practitioners and Staff Using Multilevel Mixed-Effects Logistic Regression (Physicians Only), 2018-2022

eTable 5. Estimated Probabilities of Burnout Among VHA Primary Care Practitioners and Staff Using Multilevel Mixed-Effects Logistic Regression (Physicians Only), Fiscal Years 2018-2022

eFigure 2. Estimated Probability of Burnout by Clinical Resource Hub (CRH) Implementation Percentile and Staffing Level (Physicians Only), Fiscal Years 2018-2022

eTable 6. Odds Ratios and 95% CIs of Burnout Among VHA Primary Care Practitioners and Staff Using Multilevel Mixed-Effects Logistic Regression (Nurse Practitioners Only), 2018-2022

eTable 7. Estimated Probabilities of Burnout Among VHA Primary Care Practitioners and Staff Using Multilevel Mixed-Effects Logistic Regression (Nurse Practitioners Only), Fiscal Years 2018-2022

© 2025 Apaydin EA et al. *JAMA Network Open*.

eFigure 3. Estimated Probability of Burnout by Clinical Resource Hub (CRH) Implementation Percentile and Staffing Level (Nurse Practitioners Only), Fiscal Years 2018-2022

eTable 8. Odds Ratios and 95% CIs of Burnout Among VHA Primary Care Practitioners and Staff Using Multilevel Mixed-Effects Logistic Regression (Physician Assistants Only), 2018-2022

eTable 9. Estimated Probabilities of Burnout Among VHA Primary Care Practitioners and Staff Using Multilevel Mixed-Effects Logistic Regression (Physician Assistants Only), Fiscal Years 2018-2022

eFigure 4. Estimated Probability of Burnout by Clinical Resource Hub (CRH) Implementation Percentile and Staffing Level (Physician Assistants Only), Fiscal Years 2018-2022

eTable 10. Odds Ratios and 95% CIs of Burnout Among VHA Primary Care Practitioners and Staff Using Multilevel Mixed-Effects Logistic Regression (Registered Nurses Only), 2018-2022

eTable 11. Estimated Probabilities of Burnout Among VHA Primary Care Practitioners and Staff Using Multilevel Mixed-Effects Logistic Regression (Registered Nurses Only), Fiscal Years 2018-2022

eFigure 5. Estimated Probability of Burnout by Clinical Resource Hub (CRH) Implementation Percentile and Staffing Level (Registered Nurses Only), Fiscal Years 2018-2022

eTable 12. Odds Ratios and 95% CIs of Burnout Among VHA Primary Care Practitioners and Staff Using Multilevel Mixed-Effects Logistic Regression (Clinical Associates Only), 2018-2022

eTable 13. Estimated Probabilities of Burnout Among VHA Primary Care Practitioners and Staff Using Multilevel Mixed-Effects Logistic Regression (Clinical Associates Only), Fiscal Years 2018-2022

eFigure 6. Estimated Probability of Burnout by Clinical Resource Hub (CRH) Implementation Percentile and Staffing Level (Clinical Associates Only), Fiscal Years 2018-2022

eTable 14. Odds Ratios and 95% CIs of Burnout Among VHA Primary Care Practitioners and Staff Using Multilevel Mixed-Effects Logistic Regression (Administrative Associates Only), 2018-2022

eTable 15. Estimated Probabilities of Burnout Among VHA Primary Care Practitioners and Staff Using Multilevel Mixed-Effects Logistic Regression (Administrative Associates Only), Fiscal Years 2018-2022

eFigure 7. Estimated Probability of Burnout by Clinical Resource Hub (CRH) Implementation Percentile and Staffing Level (Administrative Associates Only), Fiscal Years 2018-2022

© 2025 Apaydin EA et al. *JAMA Network Open*.

eTable 16. Odds Ratios and 95% CIs of Burnout Among VHA Primary Care Practitioners and Staff Using Multilevel Mixed-Effects Logistic Regression (Other PACT Professionals Only), 2018-2022

eTable 17. Estimated Probabilities of Burnout Among VHA Primary Care Practitioners and Staff Using Multilevel Mixed-Effects Logistic Regression (Other PACT Professionals Only), Fiscal Years 2018-2022

eFigure 8. Estimated Probability of Burnout by Clinical Resource Hub (CRH) Implementation Percentile and Staffing Level (Other PACT Professionals Only), Fiscal Years 2018-2022

This supplemental material has been provided by the authors to give readers additional information about their work.

© 2025 Apaydin EA et al. *JAMA Network Open*.

eMethods

Outcome: Burnout

Burnout was measured with two single-item subscales from the Maslach Burnout Inventory (MBI). Emotional exhaustion ("I feel burned out from my work") and depersonalization ("I worry that this job is hardening me emotionally") MBI subscales were assessed via symptom frequency on a 7-point response scale (never; a few times a year or less; once a month or less; a few times a month; once a week; a few times a week; or every day). The construct[1] and factorial[2] validity of each MBI subscale has been established using multiple samples, drawn from within the medical field and other sectors of employment, from multiple countries. We created a dichotomous measure of burnout as 1=symptoms reported once a week or more or either subscale, and 0=symptoms reported less than once a week. This use of single item scales at this frequency cut point has been validated against the full MBI subscales and several negative well-being outcomes in multiple samples,[1,3] and a produces estimates of burnout similar to those generated by the full scales as reported by a recent systematic review.[4]

Main predictors: CRH use and PCP Staffing

Annual use of CRH primary care services was measured using data from CDW,[5] and was calculated as the average number of CRH visits per 1000 primary care visits at the healthcare system-level. For each year and each healthcare system, we created an ordinal variable measuring CRH use as: none (pre-CRH implementation), low (0-33rd percentile), medium (34th-66th percentile), and high (67th-100th percentile).

We used a measure of adequacy of provider staffing at the healthcare system-level developed by O'Shea and colleagues,[6] calculated as the proportion of PCP FTE per panel of primary care patients at the clinic (1200 for physicians and 900 for NPs and PAs). Healthcare systems with values of 1.2 or greater were considered "fully staffed" and those with values less than 1.2 were considered "not fully staffed".

Covariates: Individual-level

Self-reported individual demographic covariates were taken from the AES, and included respondent age (<30 years; 30-39 years; 40-49 years; 50-59 years; 60+ years; missing), sex (male; female; missing), race/ethnicity (non-Hispanic White; non-Hispanic Black; non-Hispanic Asian; non-Hispanic Pacific Islander; Hispanic; missing), VA tenure (<1 year; 1-5 years; 6-10 years; 11-15 years; 16-20 years; 20+ years; missing), and team role (PCP; RN; LPN/LVN; administrative associate; other professionals; missing).

We also included a single-item measure of perceived workload from the AES, measured on a 5-point scale from strongly disagree to strongly agree: "My workload is reasonable." For analysis we created a dichotomous measure of reasonable workload (strongly agree, agree, and neutral vs. disagree and strongly disagree responses; missing responses also included).

© 2025 Apaydin EA et al. *JAMA Network Open*.

Covariates: Healthcare system-level

We created several healthcare system level covariates from CDW data, including PACT teamlet staffing, percent patients rural, geographic region, and healthcare system complexity.

We used a measure of core teamlet staffing assessing the proportion of primary care teams at the clinic meeting the full staffing ratio of 1 full-time equivalent (FTE) PCP to 3 FTE staff (RN, LPN/LVN, and clerk). For analysis we created a dichotomous variable for high (50% or more of teams meet 3:1 staffing ratio) vs. low staffing (less than 50% teams meet staffing ratio). The VA PACT model defines a core teamlet as 1 PCP, 1 RN, 1 clinical associate, and 1 administrative associate,[7] and so missing staff could increase the workload on PCPs delivering care within a teamlet. This variable is separate from provider staffing, which affects how patient panels and workloads are shared by PCPs between teamlets in a healthcare system. We also used healthcare system measures of percent of patients residing in a rural area and geographic region (Northeast; Midwest; South; West).

Healthcare system complexity was determined using the VA Facility Complexity Model,[8] a multi-level variable categorizing healthcare systems as 1a (most complex), 1b, 1c, 2, or 3 (least complex).[9] High complexity (Group 1a) healthcare systems usually have high risk patients, high patient volumes, the most complex clinical programs, and large teaching and research programs. Medium complexity (Groups1b and 1c) healthcare systems typically have medium-to-high risk patients, medium-to-high volume, many or some complex clinical programs, and medium to large teaching and research programs. Low complexity (Groups 2 and 3) healthcare systems are have low-to-medium volume, low risk patients, few or no complex clinical programs, and small or no research and teaching programs.

We also assessed COVID burden using a measure of COVID tests per 1000 patients at the healthcare system from the COVID Strategic Data Resource for FY 2020-22.

© 2025 Apaydin EA et al. *JAMA Network Open*.

eReferences

1. West CP, Dyrbye LN, Satele DV, Sloan JA, Shanafelt TD. Concurrent validity of single-item measures of emotional exhaustion and depersonalization in burnout assessment. *J Gen Intern Med*. Nov 2012;27(11):1445-52. doi:10.1007/s11606-012-2015-7
2. Schutte N, Toppinen S, Kalimo R, Schaufeli W. The factorial validity of the Maslach Burnout Inventory-General Survey (MBI-GS) across occupational groups and nations. *Journal of Occupational and Organizational Psychology*. 2010;73(1):53-66. doi:10.1348/096317900166877
3. West CP, Dyrbye LN, Sloan JA, Shanafelt TD. Single item measures of emotional exhaustion and depersonalization are useful for assessing burnout in medical professionals. *J Gen Intern Med*. Dec 2009;24(12):1318-21. doi:10.1007/s11606-009-1129-z
4. Rotenstein LS, Torre M, Ramos MA, et al. Prevalence of Burnout Among Physicians: A Systematic Review. *JAMA*. Sep 18 2018;320(11):1131-1150. doi:10.1001/jama.2018.12777
5. Veterans Health Administration, Health Economics Resource Center. Using the Managerial Cost Accounting 4-Character National Code (CHAR4) File for Program Evaluation. https://www.herc.research.va.gov/include/page.asp?id=using-mca-char4
6. O'Shea AMJ, Haraldsson B, Shahnazi AF, Sterling RA, Wong ES, Kaboli PJ. A Novel Gap Staffing Metric for Primary Care in the Veterans Health Administration and Implications for Rural and Urban Clinics. *J Ambul Care Manage*. Jan-Mar 01 2023;46(1):25-33. doi:10.1097/JAC.0000000000000429
7. Rosland AM, Nelson K, Sun H, et al. The patient-centered medical home in the Veterans Health Administration. *Am J Manag Care*. 2013;19(7):e263-72.
8. Veterans Health Administration. Productivity and Staffing Guidance for Specialty Provider Group Practice. Directive 1065. Washington, DC.2020.
9. National Academies of Sciences, Engineering,, Medicine. *Facilities Staffing Requirements for the Veterans Health Administration–Resource Planning and Methodology for the Future*. The National Academies Press; 2020:136.

© 2025 Apaydin EA et al. *JAMA Network Open*.

eTable 1. Odds Ratios and 95% CIs of Burnout Among VHA Primary Care Practitioners and Staff Using Multilevel Mixed-Effects Logistic Regression (Main Effects Only), Fiscal Years 2018-2022

	**N=134,640 in 139 healthcare systems**	
**Characteristic**	**OR**	**95% CI**
Ranked percentile of CRH Visits per 1,000 Primary Care Visits		
Pre-implementation	Ref	Ref
0-33 Percentile	1.14***	1.08, 1.20
34-66 Percentile	1.15***	1.10, 1.21
67-100 Percentile	1.19***	1.13, 1.25
Healthcare system-level provider staffing		
<1.2 (Not fully staffed)	1.07**	1.02, 1.12
≥1.2 (Fully staffed)	Ref	Ref
Healthcare system-level percent of core teamlets with full staffing		
<50%	Ref	Ref
≥50%	0.96*	0.93, 0.99
PACT Team Role		
Provider	Ref	Ref
RN	0.67***	0.65, 0.70
Clinical Associates	0.63***	0.60, 0.65
Administrative Associate	0.77***	0.73, 0.82
Other PACT Professionals	0.67***	0.64, 0.69
Age		
<30	Ref	Ref
30-39	0.79***	0.74, 0.84
40-49	0.63***	0.59, 0.67
50-59	0.53***	0.49, 0.56
≥60	0.42***	0.39, 0.45
Sex		
Male	Ref	Ref
Female	0.95***	0.92, 0.98
Race/Ethnicity		
Hispanic	0.99	0.95, 1.04
Non-Hispanic Asian	0.89***	0.85, 0.94
Non-Hispanic Black	0.90***	0.87, 0.94
Non-Hispanic Pacific Islander	1.03	0.90, 1.18
Non-Hispanic White	Ref	Ref
Non-Hispanic Other	1.13***	1.07, 1.22
VA Tenure		

© 2025 Apaydin EA et al. *JAMA Network Open*.

<1 year	Ref	Ref
1-5 years	1.80***	1.72, 1.88
6-10 years	2.21***	2.10, 2.31
12-20 years	2.32***	2.21, 2.44
>20 years	2.22***	2.08, 2.36
Reasonable Workload		
Neutral/Agree	Ref	Ref
Disagree	7.69***	7.47, 7.91
Facility Complexity		
1a - High	Ref	Ref
1b/c - Medium	0.95*	0.90, 0.99
2/3 - Low	1.05	0.99, 1.13
Percent of Patient Residing in Rural Area	0.999	0.998, 1.001
Census Region		
Northeast	0.87***	0.80, 0.94
Midwest	0.94	0.88, 1.01
South	0.92*	0.85, 0.99
West	Ref	Ref
Number of COVID tests per 1000 patients	1.001***	1.001, 1.002

Note: * = p<0.05, ** = p<0.01, *** = p<0.001; Full core teamlet staffing is >=3 staff per primary care provider. The "Non-Hispanic Other" group includes all respondents who self-reported a "other" race and a non-Hispanic ethnicity. Abbreviations: CI = confidence interval; OR = odds ratio; VHA = Veterans Health Administration

© 2025 Apaydin EA et al. *JAMA Network Open*.

eTable 2. Odds Ratios and 95% CIs of Burnout Among VHA Primary Care Practitioners and Staff Using Multilevel Mixed-Effects Logistic Regression (Practitioner [Physicians, Nurse Practitioners, and Physician Assistants] Only), 2018-2022

	N=22,032 in 139 healthcare systems	
**Characteristic**	**OR (95% CI)**	**p**
Ranked percentile of CRH Visits per 1,000 Primary Care Visits		
Pre-implementation	Ref	Ref
0-33 Percentile	0.90 (0.79, 1.03)	0.128
34-66 Percentile	1.07 (0.95, 1.22)	0.270
67-100 Percentile	1.20 (1.05, 1.37)	0.007
Healthcare system-level provider staffing		
<1.2 (Not fully staffed)	0.95 (0.84, 1.09)	0.461
≥1.2 (Fully staffed)	Ref	Ref
CRH Program Use x Healthcare system-level provider staffing		
Pre-implementation and not fully staffed	Ref	Ref
0-33 Percentile and not fully staffed	1.40 (1.12, 1.77)	0.003
34-66 Percentile and not fully staffed	1.22 (1.00, 1.50)	0.052
67-100 Percentile and not fully staffed	1.09 (0.88, 1.36)	0.413
Healthcare system-level percent of core teamlets with full staffing		
<50%	Ref	Ref
≥50%	0.96 (0.88, 1.03)	0.246
Age		
<30	Ref	Ref
30-39	1.00 (0.71, 1.42)	0.985
40-49	0.87 (0.61, 1.22)	0.409
50-59	0.84 (0.60, 1.19)	0.326
≥60	0.68 (0.48, 0.97)	0.031
Sex		
Male	Ref	Ref
Female	1.04 (0.97, 1.11)	0.280
Race/Ethnicity		
Hispanic	0.85 (0.75, 0.97)	0.014
Non-Hispanic Asian	0.78 (0.72, 0.85)	<.001
Non-Hispanic Black	0.73 (0.64, 0.83)	<.001
Non-Hispanic Pacific Islander	1.20 (0.81, 1.76)	0.367
Non-Hispanic White	Ref	Ref
Non-Hispanic Other	0.98 (0.80, 1.21)	0.864
VA Tenure		

© 2025 Apaydin EA et al. *JAMA Network Open*.

<1 year	Ref	Ref
1-5 years	1.64 (1.46, 1.83)	<.001
6-10 years	1.73 (1.54, 1.95)	<.001
12-20 years	1.84 (1.63, 2.08)	<.001
>20 years	1.60 (1.38, 1.85)	<.001
Reasonable Workload		
Neutral/Agree	Ref	Ref
Disagree	9.93 (9.25, 10.65)	<.001
Facility Complexity		
1a - High	Ref	Ref
1b/c - Medium	0.99 (0.90, 1.09)	0.847
2/3 - Low	1.13 (0.99, 1.28)	0.064
Percent of Patient Residing in Rural Area	1.00 (1.00, 1.00)	0.991
Census Region		
South	0.93 (0.82, 1.05)	0.235
Midwest	0.97 (0.85, 1.09)	0.571
Northeast	0.88 (0.77, 1.00)	0.051
West	Ref	Ref
Number of COVID tests per 1000 patients	1.00 (1.00, 1.00)	<.001

Note: Full core teamlet staffing is >=3 staff per primary care provider. The "Non-Hispanic Other" group includes all respondents who self-reported a "other" race and a non-Hispanic ethnicity.
Abbreviations: CI = confidence interval; OR = odds ratio; VHA = Veterans Health Administration.

© 2025 Apaydin EA et al. *JAMA Network Open*.

eTable 3. Estimated Probabilities of Burnout Among VHA Primary Care Practitioners and Staff Using Multilevel Mixed-Effects Logistic Regression (Practitioners Only), Fiscal Years 2018-2022

Ranked percentile of CRH visits per 1,000 primary care visits	Less than Full Provider Staffing *%, 95% CI*	Full Provider Staffing *%, 95% CI*	p-value for difference between predicted probabilities
**Pre-implementation**	47.2 (44.2, 50.3)	48.5 (46.2, 50.7)	0.46
**0-33 Percentile**	53.2 (48.4, 57.9)	45.9 (43.7, 48.1)	0.01
**34-66 Percentile**	54.0 (50.1, 57.9)	50.3 (48.1, 52.5)	0.09
**67-100 Percentile**	54.1 (49.8, 58.3)	53.0 (50.7, 55.3)	0.67

eFigure 1. Estimated Probability of Burnout by Clinical Resource Hub (CRH) Implementation Percentile and Staffing Level (Practitioners Only), Fiscal Years 2018-2022

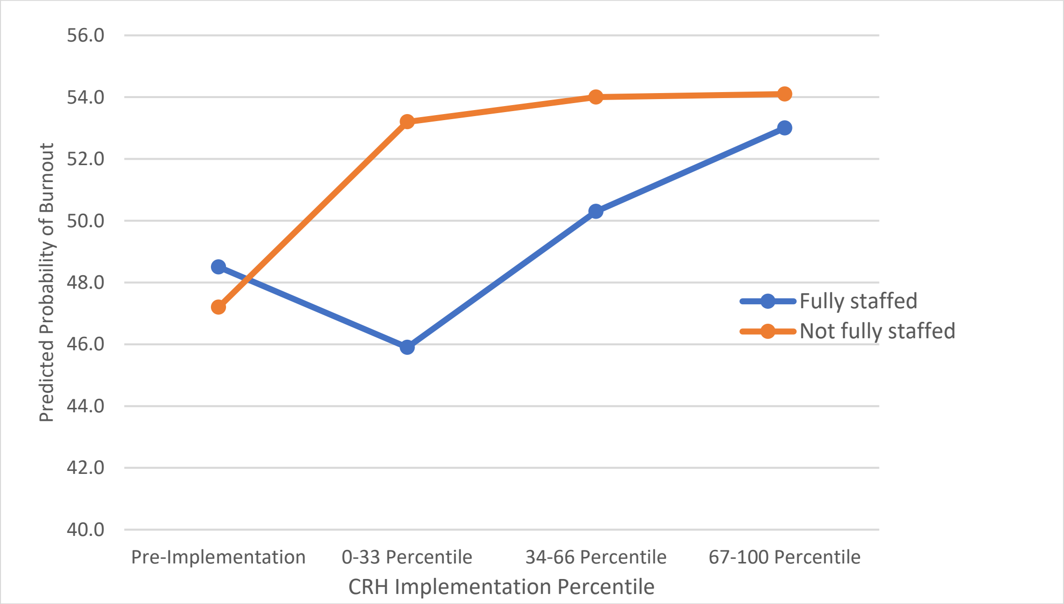

© 2025 Apaydin EA et al. *JAMA Network Open*.

eTable 4. Odds Ratios and 95% CIs of Burnout Among VHA Primary Care Practitioners and Staff Using Multilevel Mixed-Effects Logistic Regression (Physicians Only), 2018-2022

	N=15,281 in 139 healthcare systems	
**Characteristic**	**OR (95% CI)**	**p**
Ranked percentile of CRH Visits per 1,000 Primary Care Visits		
Pre-implementation	Ref	Ref
0-33 Percentile	0.92 (0.79, 1.08)	0.321
34-66 Percentile	1.06 (0.90, 1.24)	0.490
67-100 Percentile	1.19 (1.00, 1.41)	0.046
Healthcare system-level provider staffing		
<1.2 (Not fully staffed)	0.89 (0.76, 1.05)	0.161
≥1.2 (Fully staffed)	Ref	Ref
CRH Program Use x Healthcare system-level provider staffing		
Pre-implementation and not fully staffed	Ref	Ref
0-33 Percentile and not fully staffed	1.45 (1.10, 1.92)	0.008
34-66 Percentile and not fully staffed	1.35 (1.06, 1.72)	0.015
67-100 Percentile and not fully staffed	1.21 (0.92, 1.58)	0.172
Healthcare system-level percent of core teamlets with full staffing		
<50%	Ref	Ref
≥50%	0.93 (0.85, 1.03)	0.169
Age		
<30	Ref	Ref
30-39	1.28 (0.68, 2.42)	0.447
40-49	1.11 (0.59, 2.10)	0.744
50-59	1.11 (0.59, 2.10)	0.746
≥60	0.91 (0.48, 1.72)	0.766
Sex		
Male	Ref	Ref
Female	1.15 (1.07, 1.25)	<.001
Race/Ethnicity		
Hispanic	0.78 (0.67, 0.90)	0.001
Non-Hispanic Asian	0.73 (0.67, 0.81)	<.001
Non-Hispanic Black	0.70 (0.59, 0.83)	<.001
Non-Hispanic Pacific Islander	1.07 (0.66, 1.73)	0.778
Non-Hispanic White	Ref	Ref
Non-Hispanic Other	0.99 (0.76, 1.29)	0.941
VA Tenure		
<1 year	Ref	Ref

© 2025 Apaydin EA et al. *JAMA Network Open*.

1-5 years	1.52 (1.32, 1.75)	<.001
6-10 years	1.60 (1.38, 1.86)	<.001
12-20 years	1.67 (1.44, 1.94)	<.001
>20 years	1.47 (1.23, 1.75)	<.001
Reasonable Workload		
Neutral/Agree	Ref	Ref
Disagree	10.30 (9.45, 11.22)	<.001
Facility Complexity		
1a - High	Ref	Ref
1b/c - Medium	0.99 (0.87, 1.12)	0.877
2/3 - Low	1.15 (0.97, 1.37)	0.109
Percent of Patient Residing in Rural Area	1.00 (1.00, 1.00)	0.634
Census Region		
South	0.93 (0.79, 1.11)	0.436
Midwest	1.04 (0.88, 1.22)	0.646
Northeast	0.98 (0.82, 1.17)	0.807
West	Ref	Ref
Number of COVID tests per 1000 patients	1.00 (1.00, 1.00)	<.001

Note: Full core teamlet staffing is >=3 staff per primary care provider. The “Non-Hispanic Other” group includes all respondents who self-reported a “other” race and a non-Hispanic ethnicity.
Abbreviations: CI = confidence interval; OR = odds ratio; VHA = Veterans Health Administration.

© 2025 Apaydin EA et al. *JAMA Network Open*.

eTable 5. Estimated Probabilities of Burnout Among VHA Primary Care Practitioners and Staff Using Multilevel Mixed-Effects Logistic Regression (Physicians Only), Fiscal Years 2018-2022

Ranked percentile of CRH visits per 1,000 primary care visits	Less than Full Provider Staffing *%, 95% CI*	Full Provider Staffing *%, 95% CI*	p-value for difference between predicted probabilities
**Pre-implementation**	47.5 (43.6, 51.3)	50.3 (47.5, 53.2)	0.16
**0-33 Percentile**	54.8 (48.9, 60.5)	48.3 (45.6, 51.1)	0.04
**34-66 Percentile**	56.3 (51.6, 61.0)	51.7 (48.9, 54.5)	0.08
**67-100 Percentile**	56.4 (51.0, 61.6)	54.6 (51.6, 57.6)	0.56

eFigure 2. Estimated Probability of Burnout by Clinical Resource Hub (CRH) Implementation Percentile and Staffing Level (Physicians Only), Fiscal Years 2018-2022

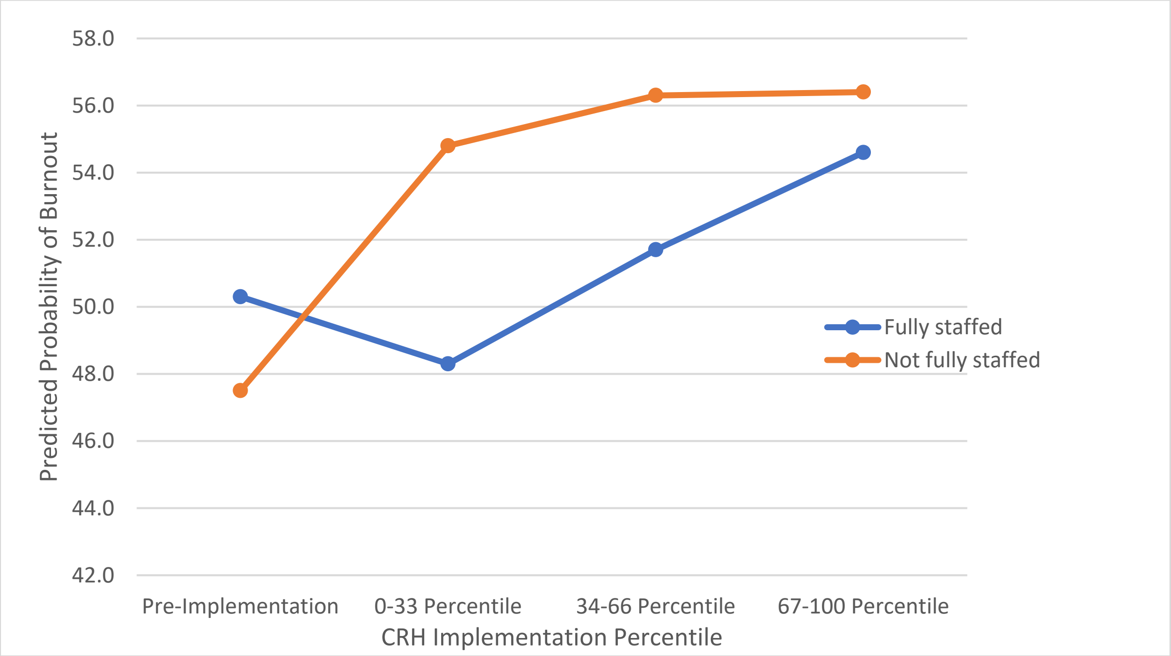

© 2025 Apaydin EA et al. *JAMA Network Open*.

eTable 6. Odds Ratios and 95% CIs of Burnout Among VHA Primary Care Practitioners and Staff Using Multilevel Mixed-Effects Logistic Regression (Nurse Practitioners Only), 2018-2022

	N=5,498 in 139 healthcare systems	
**Characteristic**	**OR (95% CI)**	**p**
Ranked percentile of CRH Visits per 1,000 Primary Care Visits		
Pre-implementation	Ref	Ref
0-33 Percentile	0.84 (0.64, 1.09)	0.193
34-66 Percentile	1.04 (0.81, 1.34)	0.753
67-100 Percentile	1.31 (1.02, 1.70)	0.037
Healthcare system-level provider staffing		
<1.2 (Not fully staffed)	1.10 (0.84, 1.43)	0.482
≥1.2 (Fully staffed)	Ref	Ref
CRH Program Use x Healthcare system-level provider staffing		
Pre-implementation and not fully staffed	Ref	Ref
0-33 Percentile and not fully staffed	1.37 (0.85, 2.23)	0.198
34-66 Percentile and not fully staffed	0.99 (0.65, 1.51)	0.964
67-100 Percentile and not fully staffed	0.77 (0.50, 1.18)	0.226
Healthcare system-level percent of core teamlets with full staffing		
<50%	Ref	Ref
≥50%	0.93 (0.80, 1.08)	0.333
Age		
<30	Ref	Ref
30-39	1.14 (0.66, 1.97)	0.648
40-49	0.94 (0.54, 1.62)	0.820
50-59	0.88 (0.51, 1.51)	0.635
≥60	0.67 (0.39, 1.17)	0.162
Sex		
Male	Ref	Ref
Female	0.99 (0.82, 1.19)	0.881
Race/Ethnicity		
Hispanic	0.87 (0.63, 1.19)	0.372
Non-Hispanic Asian	0.68 (0.53, 0.87)	0.002
Non-Hispanic Black	0.84 (0.68, 1.05)	0.119
Non-Hispanic Pacific Islander	1.67 (0.83, 3.35)	0.146
Non-Hispanic White	Ref	Ref
Non-Hispanic Other	0.95 (0.65, 1.40)	0.796
VA Tenure		
<1 year	Ref	Ref

© 2025 Apaydin EA et al. *JAMA Network Open*.

1-5 years	2.01 (1.61, 2.50)	<.001
6-10 years	2.04 (1.61, 2.58)	<.001
12-20 years	2.25 (1.76, 2.87)	<.001
>20 years	2.05 (1.53, 2.74)	<.001
Reasonable Workload		
Neutral/Agree	Ref	Ref
Disagree	9.12 (7.91, 10.51)	<.001
Facility Complexity		
1a - High	Ref	Ref
1b/c - Medium	0.98 (0.81, 1.19)	0.873
2/3 - Low	1.21 (0.96, 1.53)	0.114
Percent of Patient Residing in Rural Area	1.00 (1.00, 1.00)	0.924
Census Region		
South	0.79 (0.62, 0.99)	0.039
Midwest	0.84 (0.67, 1.05)	0.123
Northeast	0.64 (0.50, 0.82)	<.001
West	Ref	Ref
Number of COVID tests per 1000 patients	1.00 (1.00, 1.00)	<.001

Note: Full core teamlet staffing is >=3 staff per primary care provider. The "Non-Hispanic Other" group includes all respondents who self-reported a "other" race and a non-Hispanic ethnicity.
Abbreviations: CI = confidence interval; OR = odds ratio; VHA = Veterans Health Administration.

© 2025 Apaydin EA et al. *JAMA Network Open*.

eTable 7. Estimated Probabilities of Burnout Among VHA Primary Care Practitioners and Staff Using Multilevel Mixed-Effects Logistic Regression (Nurse Practitioners Only), Fiscal Years 2018-2022

Ranked percentile of CRH visits per 1,000 primary care visits	Less than Full Provider Staffing *%, 95% CI*	Full Provider Staffing *%, 95% CI*	p-value for difference between predicted probabilities
**Pre-implementation**	46.0 (39.9, 52.2)	43.7 (39.4, 48.1)	0.48
**0-33 Percentile**	49.5 (39.8, 59.2)	39.4 (35.3, 43.6)	0.06
**34-66 Percentile**	46.8 (38.9, 54.8)	44.7 (40.6, 48.9)	0.64
**67-100 Percentile**	46.3 (38.3, 54.5)	50.5 (46.3, 54.6)	0.35

eFigure 3. Estimated Probability of Burnout by Clinical Resource Hub (CRH) Implementation Percentile and Staffing Level (Nurse Practitioners Only), Fiscal Years 2018-2022

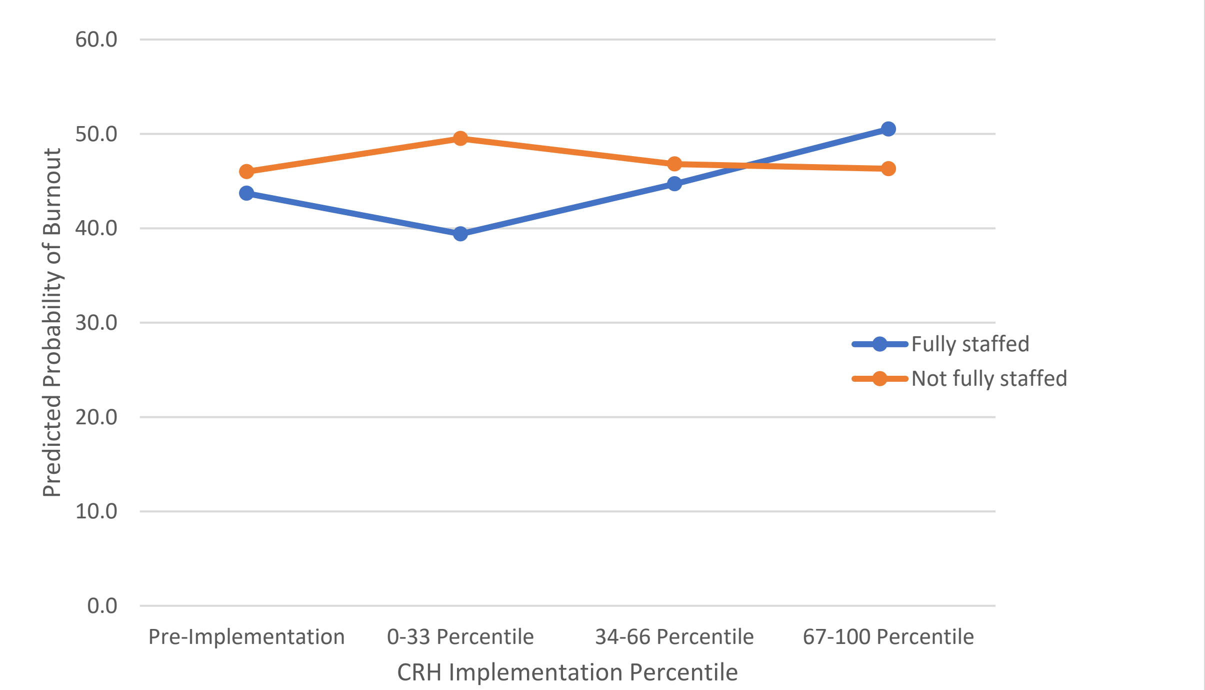

© 2025 Apaydin EA et al. *JAMA Network Open*.

eTable 8. Odds Ratios and 95% CIs of Burnout Among VHA Primary Care Practitioners and Staff Using Multilevel Mixed-Effects Logistic Regression (Physician Assistants Only), 2018-2022

	N=1,251 in 139 healthcare systems	
**Characteristic**	**OR (95% CI)**	**p**
Ranked percentile of CRH Visits per 1,000 Primary Care Visits		
Pre-implementation	Ref	Ref
0-33 Percentile	1.02 (0.55, 1.90)	0.945
34-66 Percentile	1.46 (0.80, 2.66)	0.220
67-100 Percentile	1.03 (0.58, 1.83)	0.911
Healthcare system-level provider staffing		
<1.2 (Not fully staffed)	1.07 (0.61, 1.87)	0.820
≥1.2 (Fully staffed)	Ref	Ref
CRH Program Use x Healthcare system-level provider staffing		
Pre-implementation and not fully staffed	Ref	Ref
0-33 Percentile and not fully staffed	1.73 (0.65, 4.57)	0.270
34-66 Percentile and not fully staffed	1.01 (0.41, 2.48)	0.988
67-100 Percentile and not fully staffed	1.25 (0.53, 2.95)	0.610
Healthcare system-level percent of core teamlets with full staffing		
<50%	Ref	Ref
≥50%	1.37 (0.99, 1.91)	0.061
Age		
<30	Ref	Ref
30-39	0.43 (0.20, 0.95)	0.035
40-49	0.40 (0.18, 0.86)	0.019
50-59	0.36 (0.16, 0.79)	0.010
≥60	0.29 (0.12, 0.67)	0.003
Sex		
Male	Ref	Ref
Female	0.88 (0.65, 1.19)	0.403
Race/Ethnicity		
Hispanic	0.41 (0.22, 0.75)	0.004
Non-Hispanic Asian	0.91 (0.49, 1.71)	0.769
Non-Hispanic Black	0.41 (0.22, 0.75)	0.004
Non-Hispanic White	Ref	Ref
Non-Hispanic Pacific Islander	0.97 (0.53, 1.77)	0.924
Non-Hispanic Other	0.85 (0.38, 1.92)	0.700
VA Tenure		
<1 year	Ref	Ref
1-5 years	1.27 (0.81, 1.98)	0.295

© 2025 Apaydin EA et al. *JAMA Network Open*.

6-10 years	1.93 (1.17, 3.16)	0.009
12-20 years	2.69 (1.59, 4.56)	<.001
>20 years	1.97 (0.99, 3.93)	0.053
Reasonable Workload		
Neutral/Agree	Ref	Ref
Disagree	11.62 (8.32, 16.22)	<.001
Facility Complexity		
1a - High	Ref	Ref
1b/c - Medium	1.20 (0.75, 1.93)	0.444
2/3 - Low	1.04 (0.59, 1.85)	0.881
Percent of Patient Residing in Rural Area	1.00 (0.99, 1.01)	0.826
Census Region		
South	1.13 (0.63, 2.03)	0.688
Midwest	0.68 (0.38, 1.24)	0.208
Northeast	0.69 (0.37, 1.30)	0.249
West	Ref	Ref
Number of COVID tests per 1000 patients	1.00 (1.00, 1.00)	0.798

Note: Full core teamlet staffing is >=3 staff per primary care provider. The "Non-Hispanic Other" group includes all respondents who self-reported a "other" race and a non-Hispanic ethnicity.
Abbreviations: CI = confidence interval; OR = odds ratio; VHA = Veterans Health Administration.

© 2025 Apaydin EA et al. *JAMA Network Open*.

eTable 9. Estimated Probabilities of Burnout Among VHA Primary Care Practitioners and Staff Using Multilevel Mixed-Effects Logistic Regression (Physician Assistants Only), Fiscal Years 2018-2022

Ranked percentile of CRH visits per 1,000 primary care visits	Less than Full Provider Staffing *%, 95% CI*	Full Provider Staffing *%, 95% CI*	p-value for difference between predicted probabilities
**Pre-implementation**	45.6 (33.4, 58.4)	44.0 (34.2, 54.3)	0.82
**0-33 Percentile**	59.7 (40.7, 76.2)	44.6 (34.6, 55)	0.17
**34-66 Percentile**	55.2 (38.2, 71)	53.4 (42.9, 63.6)	0.86
**67-100 Percentile**	52.0 (35.6, 68)	44.8 (35.9, 54.1)	0.44

eFigure 4. Estimated Probability of Burnout by Clinical Resource Hub (CRH) Implementation Percentile and Staffing Level (Physician Assistants Only), Fiscal Years 2018-2022

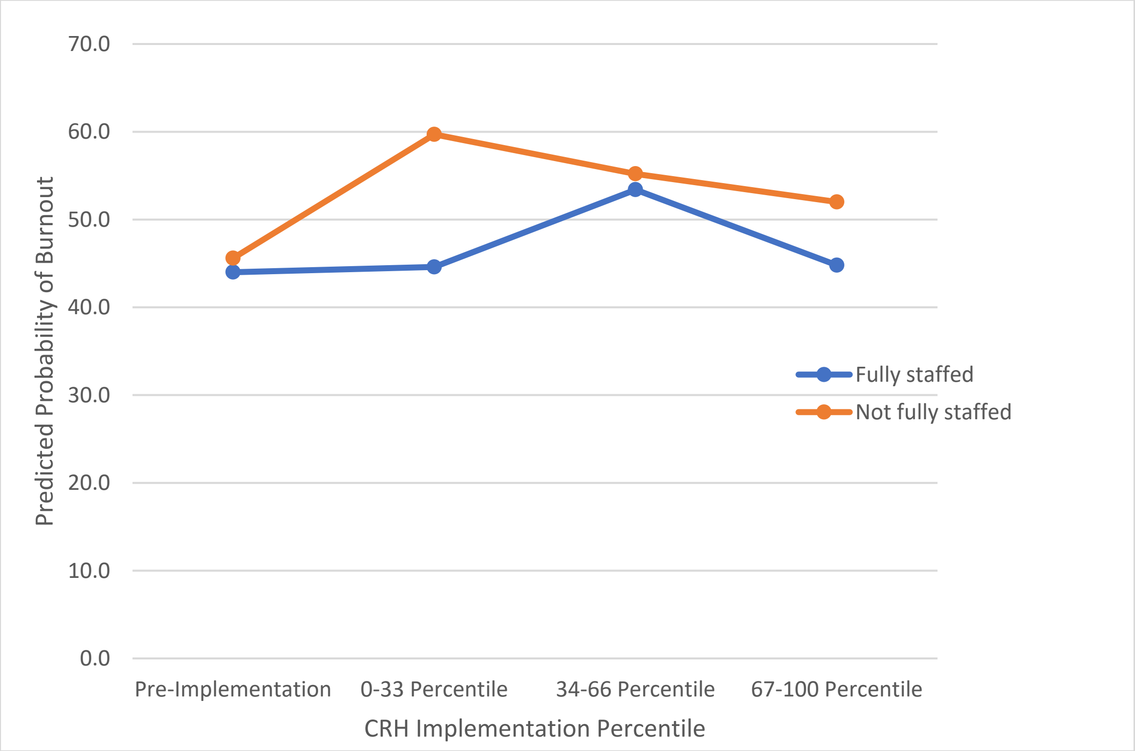

© 2025 Apaydin EA et al. *JAMA Network Open*.

eTable 10. Odds Ratios and 95% CIs of Burnout Among VHA Primary Care Practitioners and Staff Using Multilevel Mixed-Effects Logistic Regression (Registered Nurses Only), 2018-2022

	N=31,756 in 139 healthcare systems	
**Characteristic**	**OR (95% CI)**	**p**
Ranked percentile of CRH Visits per 1,000 Primary Care Visits		
Pre-implementation	Ref	Ref
0-33 Percentile	1.20 (1.07, 1.34)	0.001
34-66 Percentile	1.15 (1.03, 1.29)	0.014
67-100 Percentile	1.21 (1.08, 1.36)	0.001
Healthcare system-level provider staffing		
<1.2 (Not fully staffed)	1.09 (0.98, 1.22)	0.112
≥1.2 (Fully staffed)	Ref	Ref
CRH Program Use x Healthcare system-level provider staffing		
Pre-implementation and not fully staffed	Ref	Ref
0-33 Percentile and not fully staffed	0.96 (0.80, 1.15)	0.661
34-66 Percentile and not fully staffed	0.85 (0.72, 1.01)	0.061
67-100 Percentile and not fully staffed	0.88 (0.73, 1.06)	0.171
Healthcare system-level percent of core teamlets with full staffing		
<50%	Ref	Ref
≥50%	0.98 (0.92, 1.05)	0.581
Age		
<30	Ref	Ref
30-39	0.80 (0.68, 0.94)	0.005
40-49	0.75 (0.64, 0.87)	<.001
50-59	0.58 (0.49, 0.68)	<.001
≥60	0.46 (0.39, 0.54)	<.001
Sex		
Male	Ref	Ref
Female	0.94 (0.87, 1.01)	0.082
Race/Ethnicity		
Hispanic	0.90 (0.81, 1.00)	0.059
Non-Hispanic Asian	0.86 (0.78, 0.95)	0.003
Non-Hispanic Black	0.79 (0.73, 0.86)	<.001
Non-Hispanic Pacific Islander	0.93 (0.72, 1.21)	0.588
Non-Hispanic White	Ref	Ref
Non-Hispanic Other	1.07 (0.93, 1.24)	0.346
VA Tenure		
<1 year	Ref	Ref
1-5 years	1.96 (1.77, 2.17)	<.001

© 2025 Apaydin EA et al. *JAMA Network Open*.

6-10 years	2.35 (2.11, 2.60)	<.001
12-20 years	2.52 (2.26, 2.80)	<.001
>20 years	2.43 (2.12, 2.78)	<.001
Reasonable Workload		
Neutral/Agree	Ref	Ref
Disagree	8.32 (7.85, 8.83)	<.001
Facility Complexity		
1a - High	Ref	Ref
1b/c - Medium	0.93 (0.84, 1.02)	0.116
2/3 - Low	1.02 (0.90, 1.15)	0.760
Percent of Patient Residing in Rural Area	1.00 (1.00, 1.00)	0.451
Census Region		
South	0.91 (0.80, 1.03)	0.125
Midwest	0.93 (0.82, 1.05)	0.226
Northeast	0.88 (0.77, 1.00)	0.059
West	Ref	Ref
Number of COVID tests per 1000 patients	1.00 (1.00, 1.00)	<.001

Note: Full core teamlet staffing is >=3 staff per primary care provider. The "Non-Hispanic Other" group includes all respondents who self-reported a "other" race and a non-Hispanic ethnicity.
Abbreviations: CI = confidence interval; OR = odds ratio; VHA = Veterans Health Administration.

© 2025 Apaydin EA et al. *JAMA Network Open*.

eTable 11. Estimated Probabilities of Burnout Among VHA Primary Care Practitioners and Staff Using Multilevel Mixed-Effects Logistic Regression (Registered Nurses Only), Fiscal Years 2018-2022

Ranked percentile of CRH visits per 1,000 primary care visits	Less than Full Provider Staffing *%, 95% CI*	Full Provider Staffing *%, 95% CI*	p-value for difference between predicted probabilities
**Pre-implementation**	35.6 (33.2, 38.0)	33.6 (31.9, 35.3)	0.11
**0-33 Percentile**	38.8 (35.2, 42.6)	37.7 (35.8, 39.6)	0.58
**34-66 Percentile**	35.1 (32.0, 38.3)	36.8 (34.9, 38.7)	0.34
**67-100 Percentile**	37 (33.6, 40.6)	37.9 (36.0, 40.0)	0.64

eFigure 5. Estimated Probability of Burnout by Clinical Resource Hub (CRH) Implementation Percentile and Staffing Level (Registered Nurses Only), Fiscal Years 2018-2022

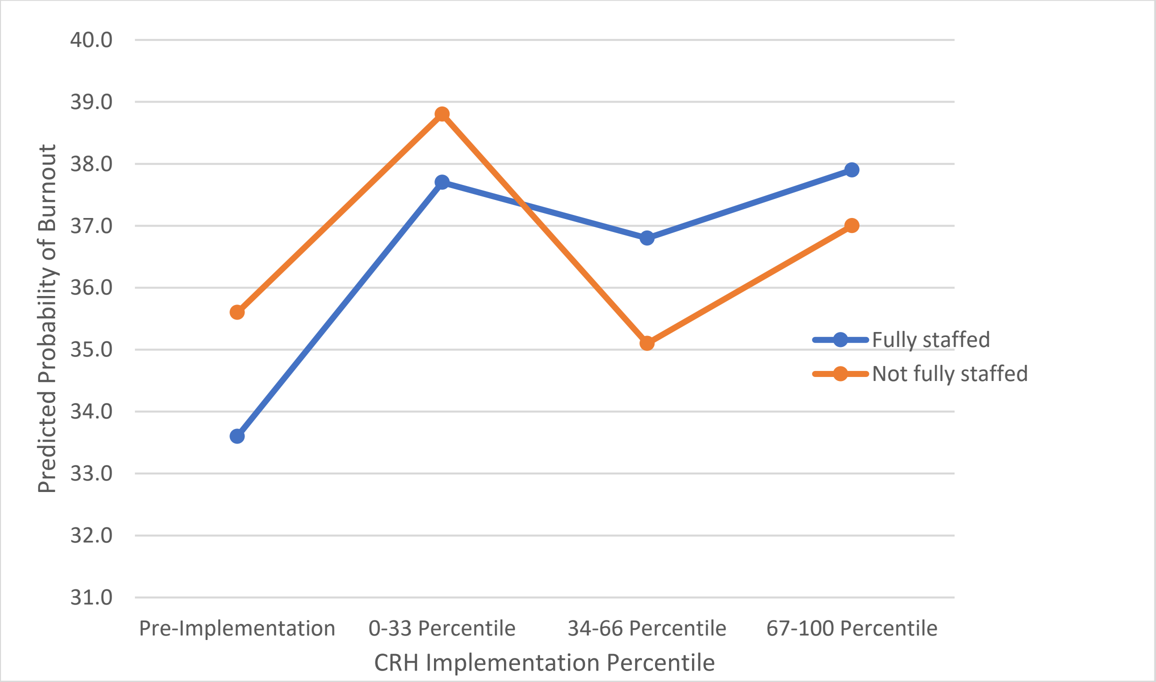

© 2025 Apaydin EA et al. *JAMA Network Open*.

eTable 12. Odds Ratios and 95% CIs of Burnout Among VHA Primary Care Practitioners and Staff Using Multilevel Mixed-Effects Logistic Regression (Clinical Associates Only), 2018-2022

	N=27,461 in 139 healthcare systems	
**Characteristic**	**OR (95% CI)**	**p**
Ranked percentile of CRH Visits per 1,000 Primary Care Visits		
Pre-implementation	Ref	Ref
0-33 Percentile	1.30 (1.15, 1.47)	<.001
34-66 Percentile	1.32 (1.17, 1.49)	<.001
67-100 Percentile	1.21 (1.07, 1.38)	0.003
Healthcare system-level provider staffing		
<1.2 (Not fully staffed)	1.14 (1.01, 1.28)	0.032
≥1.2 (Fully staffed)	Ref	Ref
CRH Program Use x Healthcare system-level provider staffing		
Pre-implementation and not fully staffed	Ref	Ref
0-33 Percentile and not fully staffed	0.87 (0.71, 1.06)	0.158
34-66 Percentile and not fully staffed	0.83 (0.69, 1.00)	0.046
67-100 Percentile and not fully staffed	0.99 (0.81, 1.20)	0.895
Healthcare system-level percent of core teamlets with full staffing		
<50%	Ref	Ref
≥50%	0.92 (0.86, 0.99)	0.034
Age		
<30	Ref	Ref
30-39	0.78 (0.68, 0.90)	<.001
40-49	0.56 (0.49, 0.65)	<.001
50-59	0.47 (0.41, 0.54)	<.001
≥60	0.40 (0.34, 0.46)	<.001
Sex		
Male	Ref	Ref
Female	0.96 (0.89, 1.04)	0.283
Race/Ethnicity		
Hispanic	0.98 (0.88, 1.09)	0.712
Non-Hispanic Asian	0.90 (0.79, 1.03)	0.121
Non-Hispanic Black	0.91 (0.84, 0.98)	0.018
Non-Hispanic Pacific Islander	0.88 (0.68, 1.14)	0.324
Non-Hispanic White	Ref	Ref
Non-Hispanic Other	1.20 (1.04, 1.37)	0.009
VA Tenure		
<1 year	Ref	Ref
1-5 years	1.83 (1.66, 2.02)	<.001

© 2025 Apaydin EA et al. *JAMA Network Open*.

6-10 years	2.43 (2.18, 2.70)	<.001
12-20 years	2.45 (2.20, 2.74)	<.001
>20 years	2.55 (2.21, 2.95)	<.001
Reasonable Workload		
Neutral/Agree	Ref	Ref
Disagree	6.98 (6.53, 7.46)	<.001
Facility Complexity		
1a - High	Ref	Ref
1b/c - Medium	0.94 (0.84, 1.04)	0.233
2/3 - Low	0.96 (0.84, 1.10)	0.546
Percent of Patient Residing in Rural Area	1.00 (1.00, 1.00)	0.098
Census Region		
South	0.87 (0.75, 1.00)	0.047
Midwest	0.85 (0.74, 0.97)	0.016
Northeast	0.83 (0.72, 0.97)	0.015
West	Ref	Ref
Number of COVID tests per 1000 patients	1.00 (1.00, 1.00)	0.277

Note: Full core teamlet staffing is >=3 staff per primary care provider. The "Non-Hispanic Other" group includes all respondents who self-reported a "other" race and a non-Hispanic ethnicity.
Abbreviations: CI = confidence interval; OR = odds ratio; VHA = Veterans Health Administration.

© 2025 Apaydin EA et al. *JAMA Network Open*.

eTable 13. Estimated Probabilities of Burnout Among VHA Primary Care Practitioners and Staff Using Multilevel Mixed-Effects Logistic Regression (Clinical Associates Only), Fiscal Years 2018-2022

Ranked percentile of CRH visits per 1,000 primary care visits	Less than Full Provider Staffing *%, 95% CI*	Full Provider Staffing *%, 95% CI*	p-value for difference between predicted probabilities
**Pre-implementation**	30.6 (28.3, 33)	28.0 (26.3, 29.8)	0.03
**0-33 Percentile**	33.2 (29.5, 37)	33.5 (31.6, 35.6)	0.86
**34-66 Percentile**	32.7 (29.5, 36.0)	34.0 (32.0, 36.0)	0.49
**67-100 Percentile**	34.6 (31.0, 38.3)	32.0 (30.0, 34.1)	0.21

eFigure 6. Estimated Probability of Burnout by Clinical Resource Hub (CRH) Implementation Percentile and Staffing Level (Clinical Associates Only), Fiscal Years 2018-2022

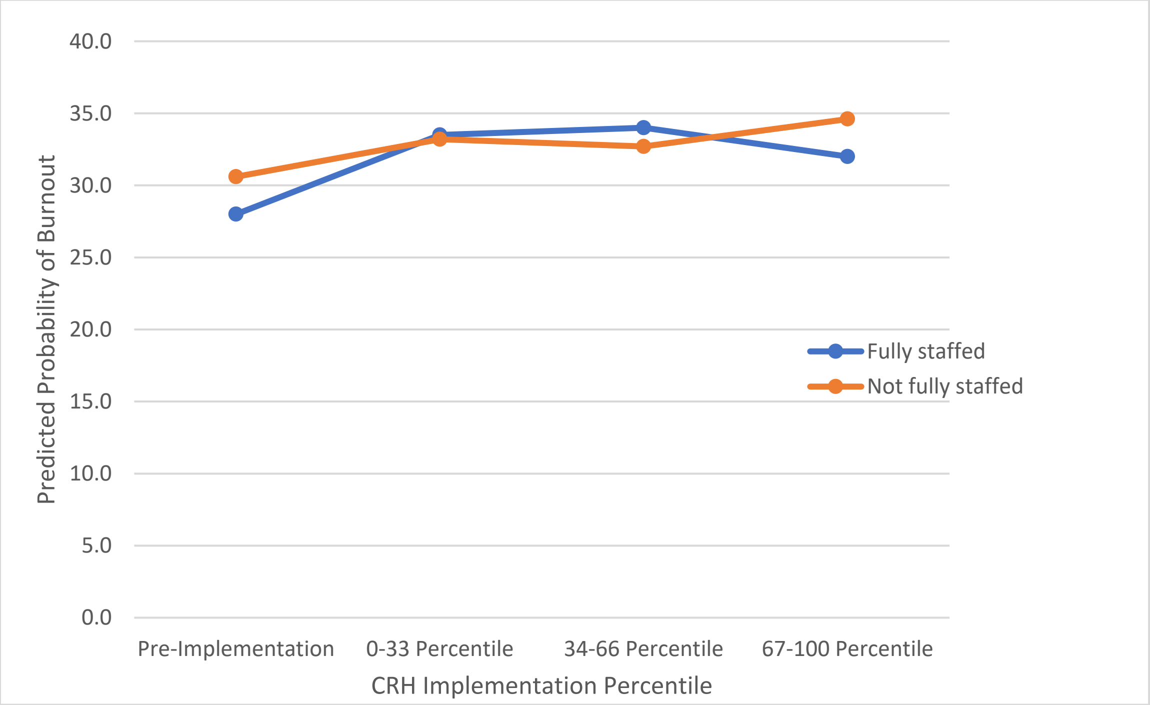

© 2025 Apaydin EA et al. *JAMA Network Open*.

eTable 14. Odds Ratios and 95% CIs of Burnout Among VHA Primary Care Practitioners and Staff Using Multilevel Mixed-Effects Logistic Regression (Administrative Associates Only), 2018-2022

	N=12,241 in 139 healthcare systems	
**Characteristic**	**OR (95% CI)**	**p**
Ranked percentile of CRH Visits per 1,000 Primary Care Visits		
Pre-implementation	Ref	Ref
0-33 Percentile	1.25 (1.05, 1.49)	0.011
34-66 Percentile	1.27 (1.07, 1.51)	0.005
67-100 Percentile	1.40 (1.17, 1.67)	<.001
Healthcare system-level provider staffing		
<1.2 (Not fully staffed)	1.23 (1.05, 1.43)	0.009
≥1.2 (Fully staffed)	Ref	Ref
CRH Program Use x Healthcare system-level provider staffing		
Pre-implementation and not fully staffed	Ref	Ref
0-33 Percentile and not fully staffed	0.84 (0.64, 1.11)	0.226
34-66 Percentile and not fully staffed	0.82 (0.63, 1.07)	0.151
67-100 Percentile and not fully staffed	0.90 (0.68, 1.18)	0.444
Healthcare system-level percent of core teamlets with full staffing		
<50%	Ref	Ref
≥50%	1.01 (0.92, 1.12)	0.764
Age		
<30	Ref	Ref
30-39	0.81 (0.68, 0.97)	0.018
40-49	0.65 (0.54, 0.77)	<.001
50-59	0.44 (0.37, 0.52)	<.001
≥60	0.32 (0.26, 0.40)	<.001
Sex		
Male	Ref	Ref
Female	0.83 (0.75, 0.91)	<.001
Race/Ethnicity		
Hispanic	0.88 (0.76, 1.01)	0.065
Non-Hispanic Asian	0.91 (0.70, 1.17)	0.447
Non-Hispanic Black	0.85 (0.76, 0.94)	0.001
Non-Hispanic Pacific Islander	1.00 (0.66, 1.50)	0.992
Non-Hispanic White	Ref	Ref
Non-Hispanic Other	1.10 (0.91, 1.32)	0.326
VA Tenure		
<1 year	Ref	Ref
1-5 years	1.76 (1.55, 1.99)	<.001

© 2025 Apaydin EA et al. *JAMA Network Open*.

6-10 years	2.07 (1.80, 2.39)	<.001
12-20 years	2.42 (2.09, 2.81)	<.001
>20 years	2.30 (1.88, 2.81)	<.001
Reasonable Workload		
Neutral/Agree	Ref	Ref
Disagree	6.59 (6.00, 7.24)	<.001
Facility Complexity		
1a - High	Ref	Ref
1b/c - Medium	0.98 (0.87, 1.10)	0.738
2/3 - Low	1.02 (0.87, 1.19)	0.803
Percent of Patient Residing in Rural Area	1.00 (0.99, 1.00)	0.014
Census Region		
South	0.92 (0.79, 1.07)	0.280
Midwest	0.96 (0.83, 1.10)	0.544
Northeast	0.85 (0.72, 1.01)	0.058
West	Ref	Ref
Number of COVID tests per 1000 patients	1.00 (1.00, 1.00)	0.557

Note: Full core teamlet staffing is >=3 staff per primary care provider. The "Non-Hispanic Other" group includes all respondents who self-reported a "other" race and a non-Hispanic ethnicity.
Abbreviations: CI = confidence interval; OR = odds ratio; VHA = Veterans Health Administration.

© 2025 Apaydin EA et al. *JAMA Network Open*.

eTable 15. Estimated Probabilities of Burnout Among VHA Primary Care Practitioners and Staff Using Multilevel Mixed-Effects Logistic Regression (Administrative Associates Only), Fiscal Years 2018-2022

Ranked percentile of CRH visits per 1,000 primary care visits	Less than Full Provider Staffing *%, 95% CI*	Full Provider Staffing *%, 95% CI*	p-value for difference between predicted probabilities
**Pre-implementation**	38.6 (35.2, 42.1)	33.9 (31.5, 36.4)	0.01
**0-33 Percentile**	39.9 (34.6, 45.4)	39.1 (36.3, 41.9)	0.80
**34-66 Percentile**	39.6 (34.7, 44.8)	39.4 (36.7, 42.3)	0.95
**67-100 Percentile**	44.1 (38.9, 49.5)	41.8 (38.8, 44.8)	0.43

eFigure 7. Estimated Probability of Burnout by Clinical Resource Hub (CRH) Implementation Percentile and Staffing Level (Administrative Associates Only), Fiscal Years 2018-2022

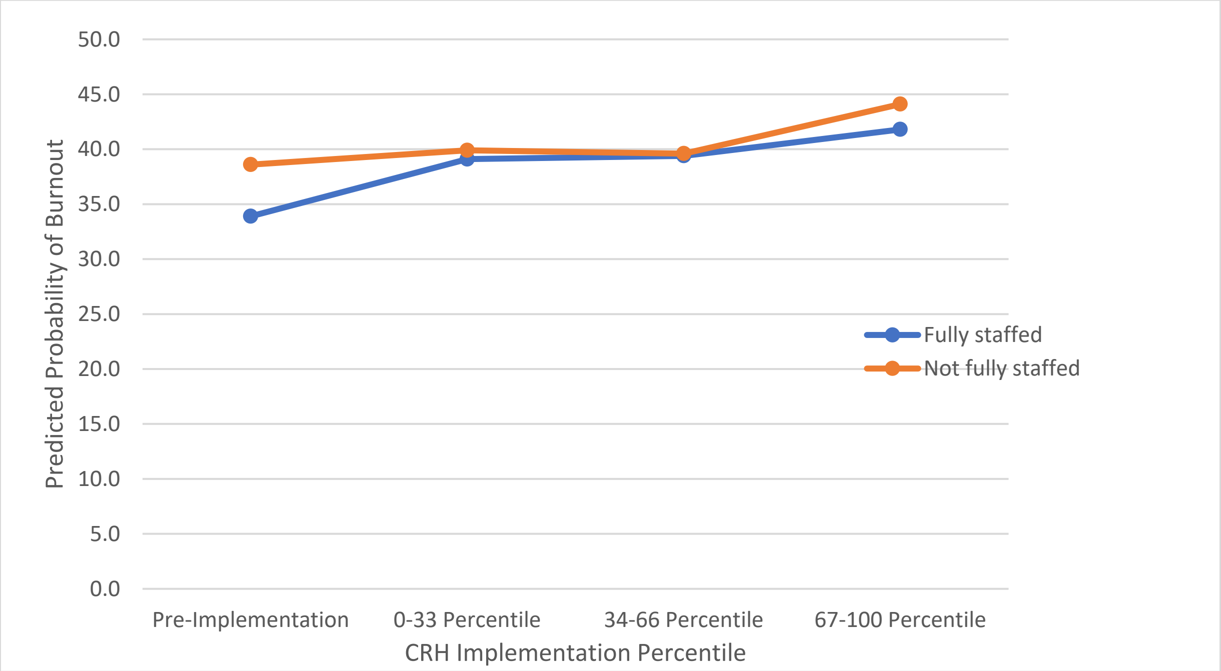

© 2025 Apaydin EA et al. *JAMA Network Open*.

eTable 16. Odds Ratios and 95% CIs of Burnout Among VHA Primary Care Practitioners and Staff Using Multilevel Mixed-Effects Logistic Regression (Other PACT Professionals Only), 2018-2022

	N=40,792 in 139 healthcare systems	
**Characteristic**	**OR (95% CI)**	**p**
Ranked percentile of CRH Visits per 1,000 Primary Care Visits		
Pre-implementation	Ref	Ref
0-33 Percentile	1.14 (1.03, 1.25)	0.010
34-66 Percentile	1.21 (1.10, 1.33)	<.001
67-100 Percentile	1.21 (1.09, 1.34)	<.001
Healthcare system-level provider staffing		
<1.2 (Not fully staffed)	1.13 (1.03, 1.24)	0.009
≥1.2 (Fully staffed)	Ref	Ref
CRH Program Use x Healthcare system-level provider staffing		
Pre-implementation and not fully staffed	Ref	Ref
0-33 Percentile and not fully staffed	1.03 (0.88, 1.21)	0.701
34-66 Percentile and not fully staffed	0.92 (0.80, 1.07)	0.289
67-100 Percentile and not fully staffed	0.87 (0.75, 1.02)	0.087
Healthcare system-level percent of core teamlets with full staffing		
<50%	Ref	Ref
≥50%	0.96 (0.91, 1.02)	0.212
Age		
<30	Ref	Ref
30-39	0.79 (0.72, 0.87)	<.001
40-49	0.60 (0.55, 0.66)	<.001
50-59	0.52 (0.47, 0.57)	<.001
≥60	0.39 (0.35, 0.43)	<.001
Sex		
Male	Ref	Ref
Female	0.95 (0.90, 1.00)	0.040
Race/Ethnicity		
Hispanic	1.19 (1.09, 1.29)	<.001
Non-Hispanic Asian	1.12 (1.01, 1.25)	0.040
Non-Hispanic Black	1.02 (0.96, 1.09)	0.507
Non-Hispanic Pacific Islander	1.40 (1.08, 1.83)	0.012
Non-Hispanic White	Ref	Ref
Non-Hispanic Other	1.23 (1.09, 1.38)	<.001
VA Tenure		
<1 year	Ref	Ref
1-5 years	1.82 (1.68, 1.96)	<.001

© 2025 Apaydin EA et al. *JAMA Network Open*.

6-10 years	2.33 (2.15, 2.54)	<.001
12-20 years	2.46 (2.26, 2.69)	<.001
>20 years	2.44 (2.16, 2.74)	<.001
Reasonable Workload		
Neutral/Agree	Ref	Ref
Disagree	6.99 (6.63, 7.37)	<.001
Facility Complexity		
1a - High	Ref	Ref
1b/c - Medium	0.91 (0.84, 0.99)	0.026
2/3 - Low	1.06 (0.96, 1.17)	0.264
Percent of Patient Residing in Rural Area	1.00 (1.00, 1.00)	0.321
Census Region		
South	0.96 (0.87, 1.07)	0.499
Midwest	1.03 (0.93, 1.14)	0.611
Northeast	0.90 (0.80, 1.00)	0.053
West	Ref	Ref
Number of COVID tests per 1000 patients	1.00 (1.00, 1.00)	<.001

Note: Full core teamlet staffing is >=3 staff per primary care provider. The "Non-Hispanic Other" group includes all respondents who self-reported a "other" race and a non-Hispanic ethnicity.
Abbreviations: CI = confidence interval; OR = odds ratio; VHA = Veterans Health Administration.

© 2025 Apaydin EA et al. *JAMA Network Open*.

eTable 17. Estimated Probabilities of Burnout Among VHA Primary Care Practitioners and Staff Using Multilevel Mixed-Effects Logistic Regression (Other PACT Professionals Only), Fiscal Years 2018-2022

Ranked percentile of CRH visits per 1,000 primary care visits	Less than Full Provider Staffing *%, 95% CI*	Full Provider Staffing *%, 95% CI*	p-value for difference between predicted probabilities
**Pre-implementation**	34.8 (32.8, 36.9)	32.1 (30.6, 33.6)	0.01
**0-33 Percentile**	38.5 (35.4, 41.7)	34.9 (33.3, 36.5)	0.04
**34-66 Percentile**	37.4 (34.7, 40.2)	36.4 (34.8, 38.0)	0.50
**67-100 Percentile**	36.1 (33.3, 39.0)	36.4 (34.7, 38.0)	0.88

eFigure 8. Estimated Probability of Burnout by Clinical Resource Hub (CRH) Implementation Percentile and Staffing Level (Other PACT Professionals Only), Fiscal Years 2018-2022

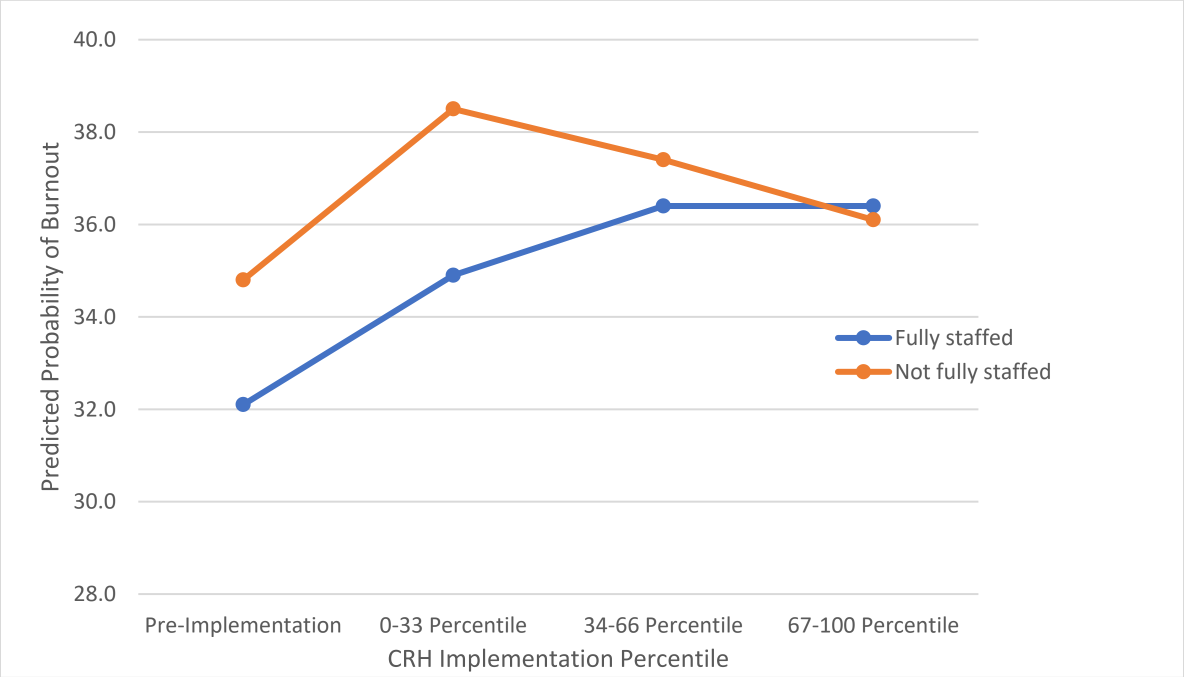

© 2025 Apaydin EA et al. *JAMA Network Open*.
