# Supplementary material for: Burnout Among Primary Care Practitioners and Staff in VA Clinics Using Virtual Contingency Staffing
Source: JAMA Netw Open. 2025 Jul 3;8(7):e2518977. doi: 10.1001/jamanetworkopen.2025.18977 (PMC12232184; doi:10.1001/jamanetworkopen.2025.18977)
Supplement: Supplement 2. — Data Sharing Statement [file jamanetwopen-e2518977-s002.pdf]

Data Sharing Statement

Apaydin. Burnout Among Primary Care Practitioners and Staff in VA Clinics Using Virtual Contingency Staffing. *JAMA Netw Open*. Published July 03, 2025. doi:10.1001/jamanetworkopen.2025.18977

Data
Data available: No

Additional Information
Explanation for why data not available: Data must remain on secure VA servers, but will be made available upon reasonable request from VA researchers.
